# Efficacy and Safety of Argatroban as an Adjunct to Antiplatelet Therapy in Ischemic Stroke: An Updated Systematic Review and Meta-analysis

**DOI:** 10.1007/s00228-026-04203-7

**Published:** 2026-10-22

**Authors:** Syeda Takreem Fatima, Ahmed Raza, Abdullah Javed, Shabnam Ijaz, Mishaal Noor, Syeda Duaa Uzair, Abdul Raheem Malik, Rana Ali Mustafa, Safi Ur Rehman Daim

**Affiliations:** 1 Department of Medicine, Services Institute of Medical Sciences, Lahore, Pakistan; 2 Department of Medicine, Allama Iqbal Medical College, Lahore, Pakistan; 3 Department of Medicine, Foundation University Medical College, Islamabad, Pakistan; 4 Department of Medicine, Rashid Latif Medical College, Lahore, Pakistan; 5 Department of Medicine, Dow University of Health Sciences, Karachi, Pakistan; 6 Department of Medicine, Continental Medical College, Lahore, Pakistan; 7 Carle Foundation Hospital, Illinois, USA

**Keywords:** Ischemic stroke, Argatroban, Antiplatelet therapy, Thrombolysis, Early neurological deterioration

## Abstract

**Introduction:**

Acute ischemic stroke (AIS) is a life-threatening condition. Argatroban can limit thrombus progression and improve AIS’s functional outcomes. This study assessed the efficacy and safety of argatroban in combination with antiplatelet therapy compared to control.

**Methods:**

PubMed and Scopus were searched. Primary outcomes were Modified Rankin Scale (mRS) 0–2 and composite hemorrhage; secondary outcomes included mRS (0–1), early neurological deterioration (END), change in National Institutes of Health Stroke Scale (NIHSS) score, mortality, intracranial and organ hemorrhage. A random-effects model was used, with risk ratios (RRs) reported for binary outcomes, while mean differences (MDs) for continuous outcomes at 95% confidence intervals (CI) and a p-value of 0.05.

**Results:**

17 studies were included with a total of 51,703 and 18,759 patients in the argatroban and control group, respectively. The primary outcomes, mRS (0–2), and composite hemorrhage showed no significant difference between argatroban and control (RR: 1.03, CI: 0.97–1.10, *p* = 0.26 and RR: 1.10, CI: 0.98–1.23, *p* = 0.11, respectively). However, argatroban significantly reduced END (RR: 0.48, CI: 0.30–0.75, *p* < 0.05) and favored mRS (0–1) with RR:1.22 (CI: 1.01–1.48, *p* = 0.03). The change in NIHSS score and incidence of mortality, stroke recurrence, intracranial and organ hemorrhage was comparable between the two groups. Subgroup analyses by antiplatelet drug in the intervention and comparison groups confirmed that argatroban showed the most pronounced results when combined with DAPT. While the overall pooled analysis did not demonstrate increased hemorrhagic risk, subgroup analysis restricted to randomized controlled trials revealed a significant increase in composite and intracranial hemorrhage with argatroban. GRADE assessment of RCT-derived evidence indicated very low to high certainty across all outcomes.

**Conclusion:**

Argatroban as an adjunct to antiplatelet therapy did not significantly improve overall functional outcome (mRS 0–2) or reduce composite hemorrhage compared with antiplatelet therapy alone. However it appears safe and may reduce END significantly.

**Supplementary Information:**

The online version contains supplementary material available at https://doi.org/10.1007/s00228-026-04203-7.

## Highlights

- Argatroban + antiplatelet therapy reduces END risk in AIS (RR: 0.48, CI: 0.30–0.75, *p* < 0.05).
- Argatroban + antiplatelet therapy improves change in NIHSS score change at discharge (MD 1.12, CI: 0.23–2.01, *p* = 0.01).
- Argatroban + antiplatelet therapy favors mRS (0–1) (RR:1.22, CI: 1.01–1.48, *p* = 0.03).
- No significant increase in the risk of composite hemorrhage with argatroban (RR: 1.10, CI: 0.98–1.23, *p* = 0.11).
- No difference in mRS 0–2 with argatroban (RR: 1.03, CI: 0.97–1.10, *p* = 0.26).

**Supplementary Information:**

The online version contains supplementary material available at https://doi.org/10.1007/s00228-026-04203-7.

## Introduction

Acute ischemic stroke (AIS) is a life-threatening condition that occurs when blood flow to the brain is cut off, causing brain tissue to become ischemic, requiring prompt treatment, which otherwise results in permanent neurological damage. Early neurological deterioration (END) is a serious complication that typically occurs within the first 48 to 72 h after the onset of AIS [1]. END is defined as a ≥ 2-point increase on the National Institutes of Health Stroke Scale (NIHSS), a reliable tool for evaluating stroke severity [2]. The reported incidence of END varies from 5% to 40% of AIS cases [2] and is associated with worse neurological outcomes, prolonged hospitalization, and increased long-term disability. Preventing END has therefore become a key therapeutic goal in AIS management.

For patients with non-cardioembolic minor stroke or large-artery atherosclerosis, dual antiplatelet therapy (DAPT), typically a combination of aspirin and clopidogrel, is a cornerstone of secondary prevention. Large randomized controlled trials have demonstrated that initiating DAPT within 24 hours significantly reduces the risk of recurrent stroke within the first 21 days [3–5]. However, prolonged use of DAPT increases the risk of gastrointestinal and intracranial bleeding, as supported by both clinical and real-world data [6, 7]. The mechanism of DAPT involves inhibition of thromboxane A2 by aspirin and suppression of ADP-mediated platelet aggregation by clopidogrel [7]. Despite its efficacy, the bleeding risks associated with DAPT warrant exploration of adjunctive or alternative strategies [8].

Argatroban is a synthetic anticoagulant that works by directly inhibiting thrombin, leading to the effective disruption of key processes, including vasoconstriction, platelet aggregation, and clot formation [9], thereby providing benefits such as enhanced blood flow through the microvasculature. In patients with progressive cerebral infarction or neurological deterioration secondary to ischemic stroke, there is an increase in the activity of coagulation and fibrinolytic systems, suggesting that the potential use of anticoagulants like argatroban in addition to antiplatelet might prove to be beneficial in improving neurological outcomes [10]. Owing to its short half-life and reversibility, it is considered favorable for acute settings where precise coagulation control is necessary [11]. Early results from pilot and observational studies suggest that argatroban combined with antiplatelet therapy may reduce the risk of END and improve functional outcomes without significantly raising the risk of bleeding [11].

A previous systematic review and meta-analysis [12] has concluded that, in comparison to antiplatelets alone, argatroban and antiplatelet therapy improve the functional outcomes and decrease END. With the emergence of new studies [13, 14] on the same topic, this systematic review and meta-analysis aims to provide an updated collection of the available evidence on the efficacy and safety of argatroban combined with antiplatelet therapy in AIS.

## Methods

This updated systematic review and meta-analysis was performed in accordance with the Preferred Reporting Items for Systematic reviews and Meta-Analyses (PRISMA) guidelines [15] and has been registered with PROSPERO (PROSPERO ID: CRD420251117369). Additionally, the AMSTAR-2 tool [16] was also used for the critical appraisal of the systematic review.

### Data sources and search strategy

A systematic search was conducted through Medline (PubMed), Scopus and Google Scholar from inception to June 16, 2025, without language restrictions, to identify relevant studies. We combined both medical subject headings (MeSH) and free-text terms to search for potential studies. The search strings for the above databases have been shown in the Supplementary Table 1.

### Eligibility criteria and study selection

The references of the extracted publications were carefully reviewed for relevant studies. All relevant articles were exported to Rayyan AI [17], where duplicates were identified and removed. All remaining studies were screened and shortlisted by two reviewers (S.T.F. and A.R.) based on their relevance to the inclusion criteria. All discrepancies were resolved by a third reviewer (A.J.). The inclusion criteria included randomized controlled trials (RCTs) and non-randomized studies (NRSs) comparing argatroban combined with antiplatelet therapy versus antiplatelet therapy alone among patients with AIS.

### Data items and extraction

Data extracted included study design, author name, publication year, sample size, patient characteristics, intervention details, comparator groups, and reported outcomes. The primary efficacy outcome included Modified Rankin Scale (mRS) 0–2, while the primary safety outcome was composite hemorrhage. The secondary outcomes were mRS (0–1), early neurological deterioration (END), change in NIHSS score, mortality, intracranial and organ hemorrhage, and stroke recurrence. mRS covers the entire range of functional outcomes in stroke patients, with a score of 0 corresponding to no symptoms, while a score of 6 corresponds to death [18]. Composite hemorrhage was defined as the occurrence of any hemorrhagic complication (including intracranial, extracranial, organ, or other bleeding events). Since intracranial hemorrhage is the most feared complication of antiplatelet therapy [19], it was analyzed separately as well. END was predominantly defined as ≥ 2 points in the NIHSS score within 7 days of hospitalization. NIHSS score is a widely used numerical scale that assesses stroke severity. A higher score indicates poor motor and sensory functionality [20]. NIHSS data were consistently extracted as change-from-baseline values across all contributing studies. This was calculated as baseline NIHSS minus the NIHSS reported at the relevant follow-up timepoint. The follow-up timepoint varied from discharge to 7 days to 90 days among the included studies (Supplementary Table 2).

### Risk of bias assessment

Risk of bias was assessed using the RoB 2 tool for RCTs [21] and the ROBINS-I tool for NRSs [22].

### Data synthesis and statistical analysis

Data was analysed with R (version 4.5.0) using the meta package (version 8.0.2). A random-effects model was used to pool effect estimates, accounting for anticipated heterogeneity between studies. For dichotomous outcomes, the Mantel-Haenszel method [23] was used, while continuous outcomes were analysed using the generic inverse variance method. Between-study variance (τ²) was estimated using the restricted maximum likelihood (REML) method [24], which was applied by default in the R meta package. Effect sizes were reported as risk ratios (RRs) with 95% confidence intervals (CIs) for binary outcomes, and mean difference (MDs) for continuous neurological outcome, change in NIHSS score from baseline to the reported follow-up assessment. When studies directly reported the mean change in NIHSS score and its corresponding SD, these values were used preferentially. For studies that reported NIHSS scores separately at baseline and follow-up but did not provide the mean change, the mean change was calculated as the baseline mean minus the follow-up mean. When the SD of the change score was not available, it was derived from the baseline and follow-up SDs using the standard formula:

$$\\\begin{array}{c}SD\;of\;Change\;=\\\surd(SD\;baseline^2\;+\;SD\;follow-up^2-\\\;2r\times SD\;baseline\;\times\;SD\;follow-up)\end{array}$$

where r represents the within-participant correlation between baseline and follow-up NIHSS measurements. In the primary analysis, a correlation coefficient of *r* = 0.50 was assumed when the required correlation was unavailable. Statistical heterogeneity was assessed using the I² statistic and the Chi-square test, with I² values greater than 50% indicating substantial heterogeneity. Leave-one-out sensitivity analysis was performed to assess the robustness of the results. Subgroups based on the antiplatelet drugs used along with argatroban in the intervention group included: argatroban and DAPT [13, 14, 25–28], argatroban and SAPT [9, 11, 29, 30], argatroban alone [31–34], and argatroban and SAPT or DAPT [35–37]. Similarly, subgroups based on the drug used in the comparison group included: DAPT [9, 11, 13, 14, 25–28], SAPT [27, 30, 34], SAPT or DAPT [33, 35–37], and standard therapy [31, 32]. Subgroup analyses according to the intervention and comparison arms, risk of bias assessment, study design and stroke phenotype were performed. Because included studies reported NIHSS score change at markedly different post-treatment timepoints, ranging from hospital discharge to 7 days to 90 days, we performed a formal subgroup analysis stratifying NIHSS change by assessment timepoint. Publication bias was assessed using Egger’s test and trim and fill analysis for the outcome mRS (0–2), which was the only outcome reported by ≥ 10 studies [38, 39]. GRADE assessment was performed to evaluate the certainty of evidence for outcomes in the RCT-only subgroup [40].

## Results

### Search results and study selection

**Fig. 1:**
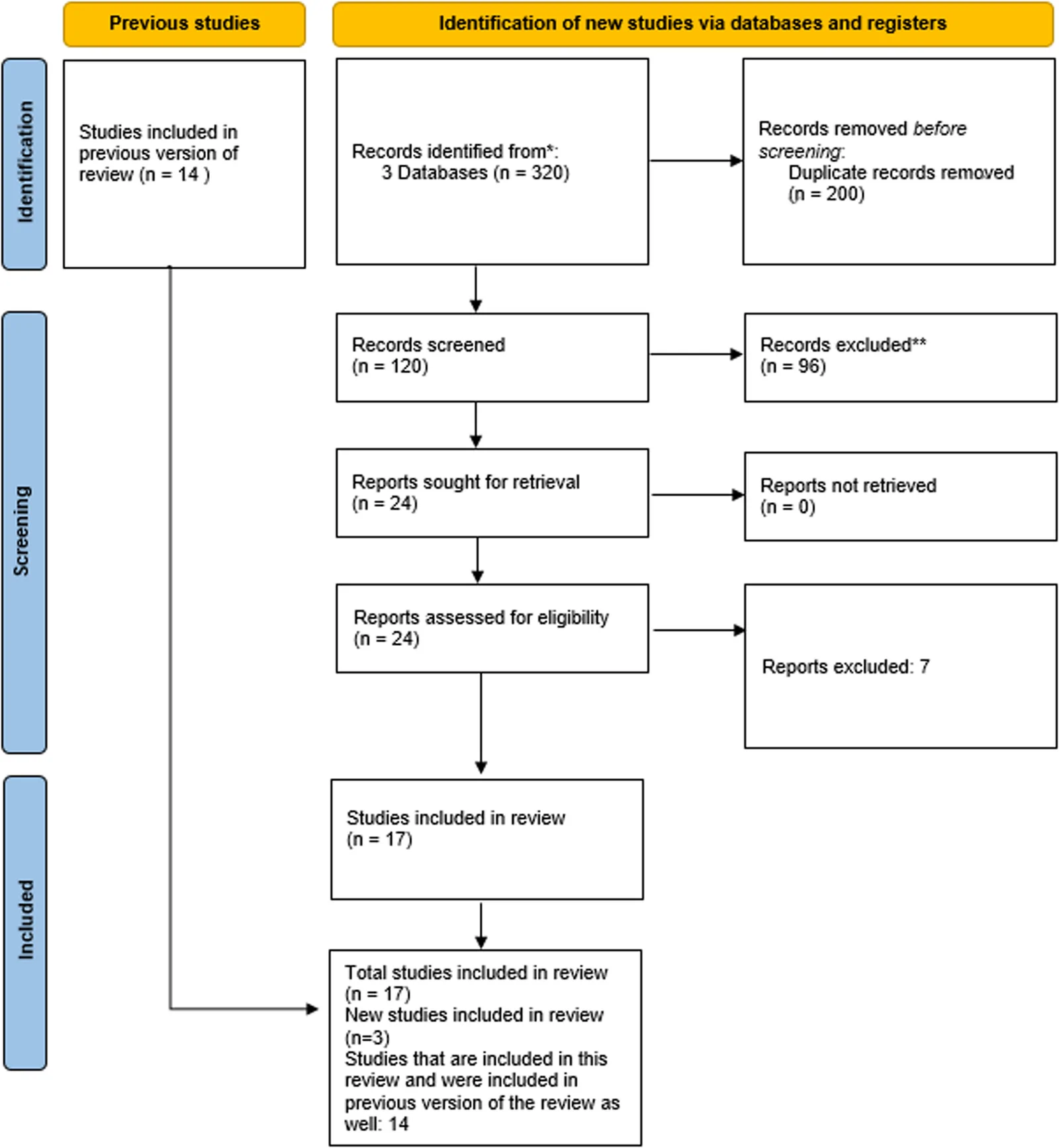
PRISMA flow diagram

The search process of the databases resulted in a total of 360 articles, including 200 duplicates. After the articles’ title and abstract screening, 24 were eligible for full-text screening. Finally, 3 new studies [13, 14, 25] and 14 studies [9, 11, 26–37] from the previous systematic review and meta-analysis were included in this review (Fig. 1). The references of extracted studies were carefully reviewed to search relevant studies that could be included in this review. This search did not give us any studies.

### Study characteristics

The included studies comprised RCTs = 6 and cohort studies = 11. Eight studies [9, 11, 13, 25–28] compared argatroban plus SAPT or DAPT with DAPT alone. Three studies [29–31] investigated argatroban plus SAPT versus SAPT, while four others [32, 35–37] evaluated argatroban plus SAPT or DAPT against SAPT or DAPT alone. One retrospective cohort study [33] compared argatroban to ozagrel. Adeoye et al. [34] assessed argatroban versus eptifibatide alone. Argatroban regimens typically involved an initial daily dose of 60–120 mg administered intravenously for the first two days, often followed by a reduced maintenance dose (10–30 mg daily) over several days. When used, clopidogrel was most commonly started with a loading dose of 300 mg followed by 75 mg daily, alongside aspirin at a standard dose of 100 mg per day. The timing of administration ranged from within 6 h to up to 72 h after stroke onset. The details of the baseline characteristics of the included studies are provided in Table 1.

**Table 1:** Baseline characteristics of the included studies

Author name (Year)	Comparison groups		Design	Population (type of stroke)	Dose		Timing of intervention	Sample size, *n*		Age, mean (SD)		Male, *n* (%)	
	Argatroban	Control group			Argatroban	Control		Argatroban	Control	Argatroban	Control	Argatroban	Control
Nakashima 2025	Argatroban + DAPT	DAPT	Retrospective cohort study	Acute atherothrombotic stroke	60 mg/day for two days. 10 mg BID for 5 days.	NA	Within 48 h	47,213	12,770	73 (12)	73 (12)	30,086 (63.7)	8,093 (63.4)
Xu 2025	Argatroban + DAPT	DAPT	RCT	Branch atherosclerosis disease	60 mg/day for 2 days 20 mg/day for next 5 days + DAPT	1 st day: 100 mg aspirin + 300 mg clopidogrel Next 3 weeks: 100 mg aspirin + 75 mg clopidogrel	Within 48 h	49	51	62 (12.9)	66.67 (14.07)	33 (67.3)	30 (58.8)
Zhang 2025	Argatroban + DAPT	DAPT	Retrospective cohort study	Lacunar stroke	120 mg/d for day 1 & 2.20 mg/d for next days	Aspirin 100 mg/d + Clopidogrel 75 mg/d	Immediately	30	50	65.1 (9.50)	64.7 (10.2)	18 (60)	29 (58)
Yan 2025	Argatroban + SAPT or DAPT	SAPT or DAPT	Retrospective cohort study	Acute mild to moderate ischemic stroke with large artery atherosclerosis	Argatroban: 60 mg/day IV infusion for 2 days, then 10 mg IV over 3 h, twice daily for 5 days	Aspirin 100 mg/day, Clopidogrel 75 mg/day, or both	Within 72 h of symptom onset	294	289	68.1 (10.8)	71.2(11.4)	195 (66.3)	172 (59.5)
Jin 2024	Argatroban + Aspirin	DAPT	Retrospective cohort study	Minor ischemic stroke	Argatroban 60 mg/day infusion for 48 h, followed by 10 mg infusion over 3 h twice daily for 5days	Aspirin:100mg/day combined with clopidogrel 75 mg/day, clopidogrel loading dose 300 mg	Within 24 h	207	207	67.6 (10.3)	67.6 (13.3)	71 (34.3)	74 (35.7)
Adeoye 2024	Argatroban	Eptifibatide	RCT	Acute ischemic stroke	Argatroban: 100 µg/kg IV bolus, then 3 µg/kg/min infusion over 12 h.	Eptifibatide: 135 µg per kilogram followed by a 2-hour infusion (0.75 µg per kilogram per minute)	Argatroban started within 75 min after thrombolysis (within 3 h of stroke onset)	59	228	69 (14)	67.3 (14.8)	31 (53)	118 (52)
Sun 2024	Argatroban + Aspirin or Clopidogrel	Aspirin or Clopidogrel	Retrospective cohort study	Acute ischemic stroke	Argatroban IV: 60 mg/day for 2 days, then 20–30 mg/day for 2–5 days - Aspirin 100 mg/day or Clopidogrel 75 mg/day	Aspirin: 100 mg/day or Clopidogrel: 75 mg/day	Within 72 h of stroke onset	57	56	58.7 (10.3)	58.3 (10.5)	26 (45.61)	27 (48.21)
Zhang 2024	Argatroban + SAPT or DAPT	SAPT or DAPT	RCT	Acute ischemic stroke	Argatroban: 60 mg/day IV infusion for 2 days, then 20 mg/day for 5 days + mono or dual antiplatelet therapy	Standard mono or dual antiplatelet therapy (aspirin and/or clopidogrel)	Within 48 h of symptom onset	302	307	65.6 (12.5)	65.3 (13.3)	191 (63.2)	195 (63.5)
Li 2022	Argatroban + DAPT	DAPT	Prospective cohort study	Acute mild to moderate ischemic stroke with large artery atherosclerosis	Argatroban: 100 µg/kg IV bolus over 3–5 min, then 1.0 µg/kg/min continuous infusion for 2–5 days (adjusted to APTT 1.75× baseline ± 10%) Clopidogrel: 300 mg loading dose, then 75 mg/day Aspirin: 100 mg/day	Clopidogrel: 300 mg loading dose, then 75 mg/day Aspirin: 100 mg/day	Within 48 h of onset	120	529	60.9 (8.5)	61.7 (9.8)	88 (73.3)	380 (71.8)
Wang 2021	Argatroban +Aspirin OR Clopidogrel	DAPT	Retrospective cohort study	Branch atherosclerosis disease	Argatroban 60 mg/day at day 1 & 2, and then twice a day (20 mg/day) for 5 days	Aspirin(100 mg/day) and clopidogrel: an initial dose of 300 mg, followed by 75 mg/day for 3 wk	Within 6–12 h of symptom onset	136	168	63 (6.4)	62 (7.8)	73 (59.3)	96 (57.1)
Xu 2021	Argatroban + DAPT	DAPT	Retrospective cohort study	Branch atherosclerotic disease	Argatroban: 60 mg/d for 2 days. 20 mg/d for the next 5 days. DAPT: Clopidogrel + Aspirin	DAPT: Clopidogrel 300 mg on day 1 followed by 75 mg/d + Aspirin 100 mg/d	Within 48 h of onset	25	55	61.6 (18.5)	63.3 (12.5)	14 (56.0)	41 (74.5)
Zhou 2020	Argatroban + DAPT	DAPT	Retrospective cohort study	Acute Minor Posterior Circulation Ischemic Stroke	Continuous infusion with a dose of 60 mg/d for 2 days, followed by 20 or 30 mg per day for 2 to 5 days	Clopidogrel: an initial dose of 300 mg, followed by 75 mg daily plus 100 mg aspirin daily	Within 48 h	34	68	61.7 (9.8)	61.7 (10.9)	25 (73.5)	49 (72.1)
Liu 2020	Argatroban + SAPT or DAPT	SAPT or DAPT	RCT	Acute paraventricular ischemic stroke	Argatroban: 10 mg IV twice daily for 7 days	Standard therapy: Antihypertensives, antiplatelets, statins	Within 48 h	30	30	57.7 (8.8)	57.1 (10.7)	23 (77)	24 (80)
Oguro 2018	Argatroban	Ozagrel	Retrospective cohort study	Acute ischemic stroke	Argatroba:"120 mg/day infusion over 2 h twice daily in the morning and the evening for 2 d, followed by 20 mg/d for 5 days	Ozagrel: 80 mg drips over 2 h twice daily, in the morning and the evening	Within 48 h	353	160	75 (12.5)	73.6 (12.5)	210 (59.5)	99 (61.9)
Chen 2018	Argatroban + Aspirin	Aspirin alone	Retrospective cohort study	Acute ischemic stroke	Argatroban: 60 mg/day IV for 2 days, then 20 mg/day for up to 4 weeks Aspirin: 100 mg/day orally	Aspirin: 300 mg/day orally	Within 48 h of stroke onset	434	1051	65.5 (10.8)	65.1(11.1)	283 (65.2)	710 (67.6)
Wada 2015	Argatroban	Standard therapy	Retrospective cohort study	Atherothrombotic stroke	Argatroban: 60 mg/day for day 1 & 2, followed by twice daily infusion, at a dose of 10 mg over 3 h, for 5 days.	NA	Within 24 h	2289	2289	40 (85)	40 (85)	1457 (63.7)	1443 (63)
LaMonte 2004	Argatroban	Placebo	RCT	Acute Ischemic Stroke	Initial 100-g/kg bolus over 3 to 5 min, followed by continuous infusion at 3 µg/kg/min for 5 days	Aspirin 81 to 325 mg for 5 days	≤ 12 h from onset	59	54	70 (13)	65 (13)	28 (47)	23 (42.6)

*DAPT* Dual Antiplatelet Therapy, *SAPT* Single Antiplatelet Therapy, *RCT* Randomized Controlled Trial, *NA* Not Available

### Risk of bias in included studies

After evaluation using Rob-2, five out of six RCTs were found to have a low risk of bias, while one had some concerns (Supplementary Fig. 1). According to ROBINS-1, which was used for cohort studies, eight studies were found to have a serious risk of bias, while three of them had a moderate risk of bias (Supplementary Fig. 2).

### Synthesis of results

#### Primary outcomes

##### Modified Rankin Scale (mRS) 0–2

**Fig. 2:**
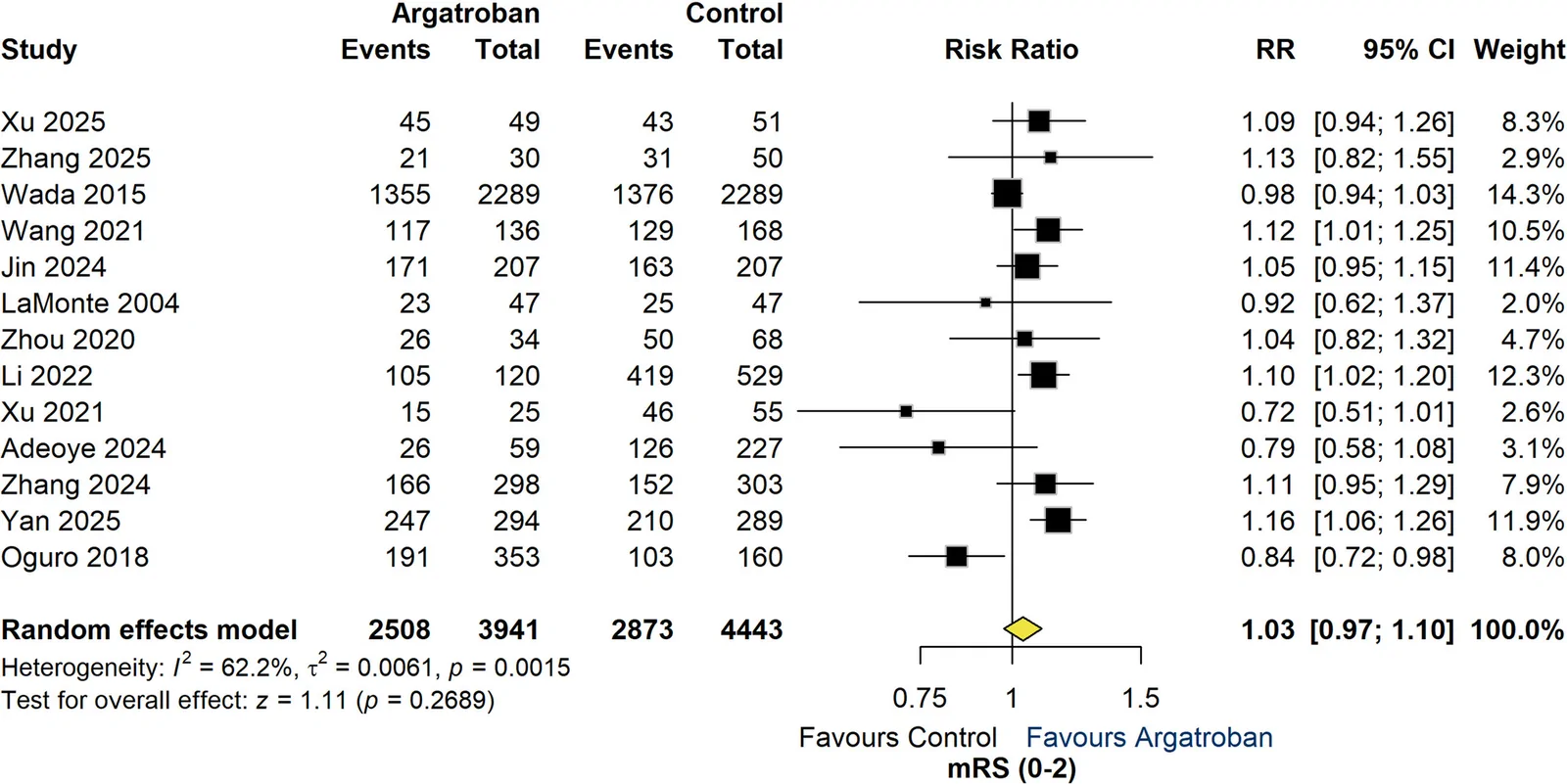
Forest plot showing the pooled RR of mRS (0–2) for argatroban vs. control

A total of 13 out of 17 studies reported mRS (0–2), with 3,941 patients being treated with argatroban and 4,443 patients receiving control. The pooled analysis showed no statistically significant difference between argatroban and control in achieving an increased incidence of mRS (0–2), with an RR of 1.03 (95% CI: 0.97–1.10; *p* = 0.26) and I^2^ = 62.2% (Fig. 2). Leave-one-out analysis, omitting Oguro et al. [33], reduced the heterogeneity to I^2^ = 54%, p = 0.02 (Supplementary Fig. 3). To evaluate the potential for publication bias, Egger’s regression test and the Trim and Fill method were applied. Egger’s test yielded a non-significant result (t = −0.44, df = 11, p = 0.66), indicating no statistically significant evidence of small-study effects or funnel plot asymmetry. Complementarily, the trim-and-fill analysis suggested the presence of one potentially missing study. (Supplementary Figs. 4 and 5).

##### Composite Hemorrhage

**Fig. 3:**
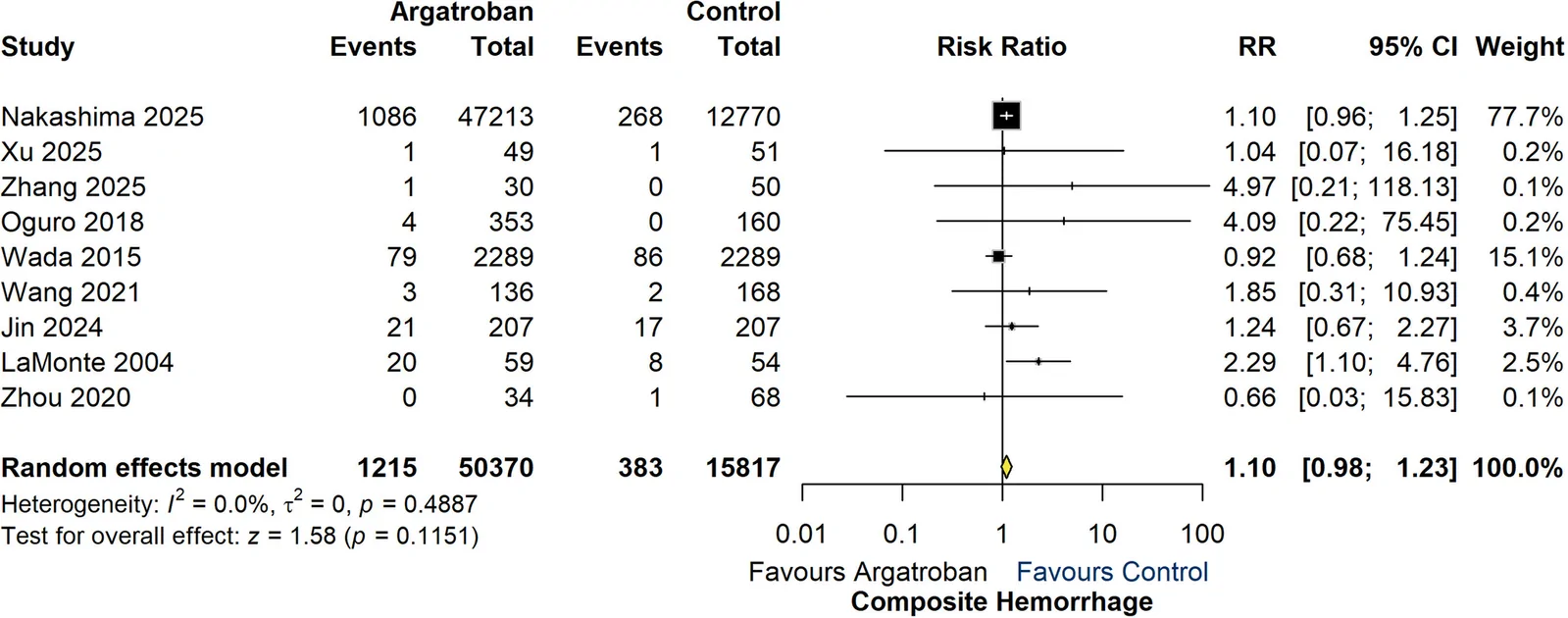
Forest plot showing the pooled RR of composite hemorrhage for argatroban vs. control

The pooled results of 9 studies (argatroban: 50,370; control: 15,817 patients) revealed no statistically significant difference in the risk of composite hemorrhage between the argatroban and control groups (RR: 1.10; 95% CI: 0.98–1.23; *p* = 0.11, I² = 0.0%; Fig. 3).

#### Secondary outcomes

##### mRS (0–1)

**Fig. 4:**
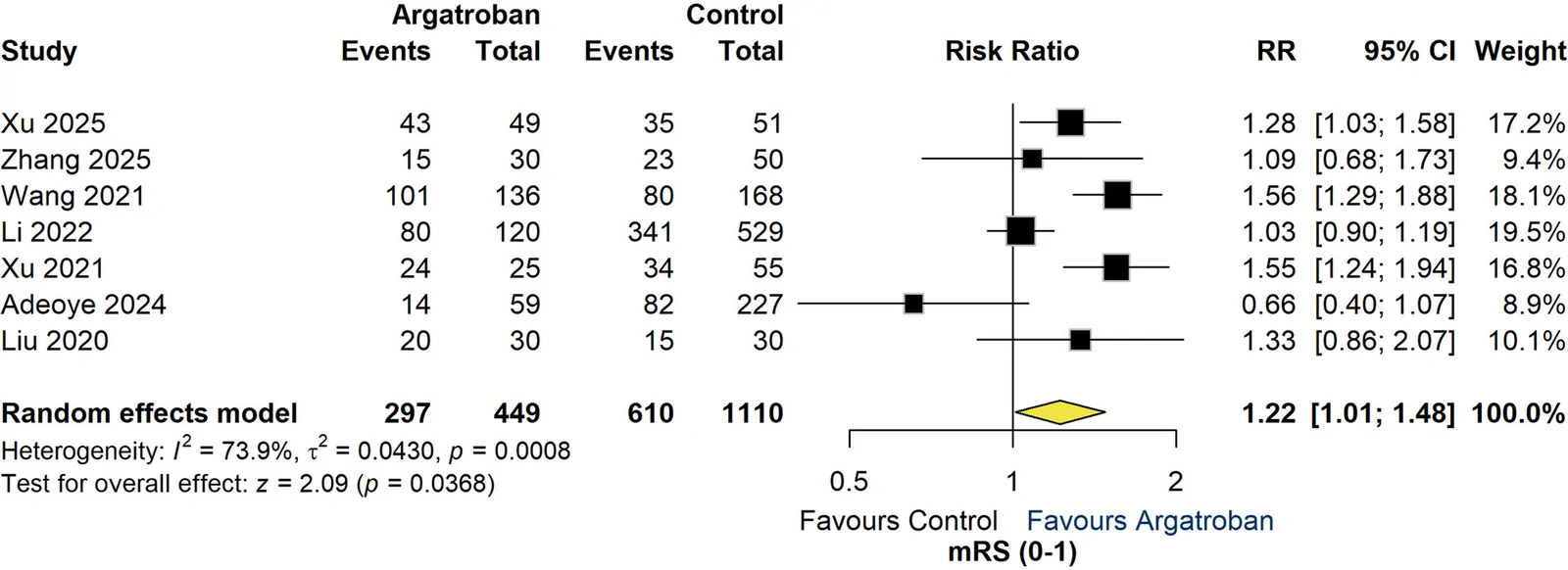
Forest plot showing the pooled RR of mRS (0–1) for argatroban vs. control

The argatroban group, with 449 total patients, when compared to the control group, with 1,110 total patients, demonstrated a statistically significant increased incidence of mRS (0–1) with RR of 1.22 (95% CI: 1.01–1.48, *p* = 0.03, I² = 73.9%; Fig. 4). Leave-one-out analysis revealed that omitting Li et al. [27] yielded statistically significant results (Supplementary Fig. 6).

##### Risk of Early Neurological Deterioration

**Fig. 5:**
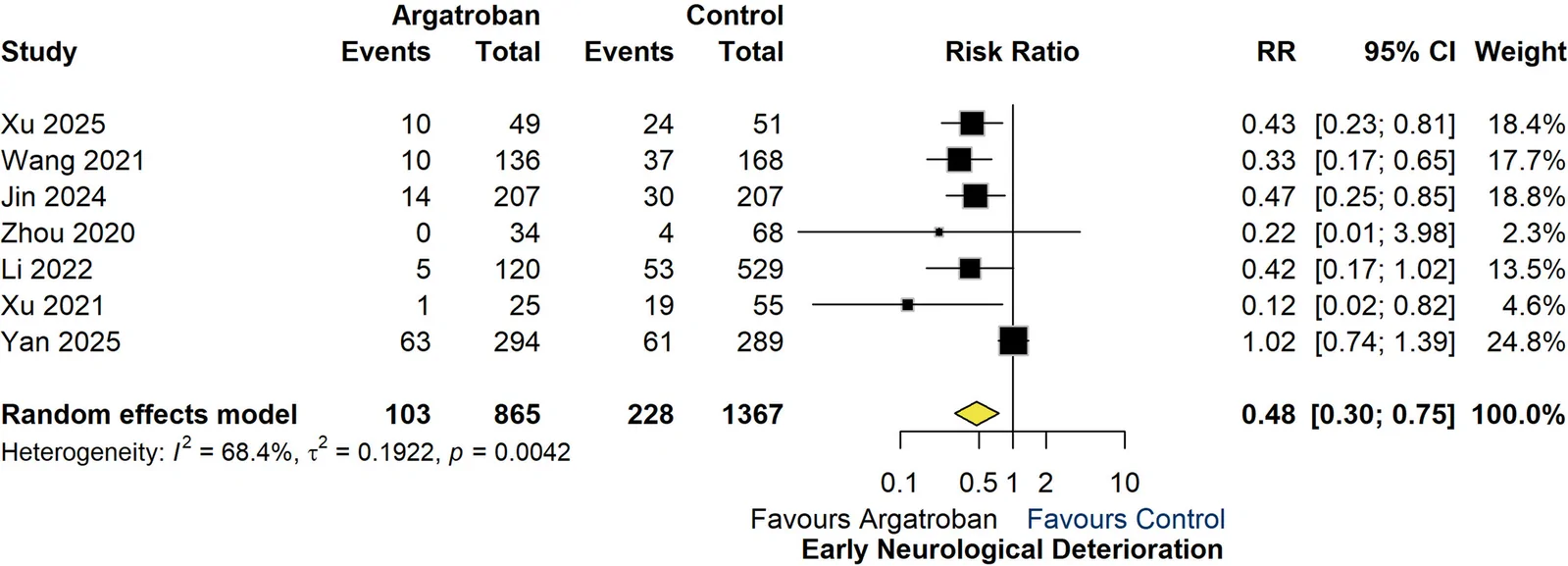
Forest plot showing the pooled RR of END for argatroban vs. control

With a total of 865 patients in the argatroban group versus 289 in the control group, argatroban significantly reduced the risk of END compared to control treatment (RR: 0.48, 95% CI: 0.30–0.75, *p* < 0.05, I^2=^ 68.4%; Fig. 5). The heterogeneity was resolved when the study by Yan et al. [35] was excluded during leave-one-out analysis (I² = 0%) (Supplementary Fig. 7).

##### Change in National Institutes of Health Stroke Scale (NIHSS) score

**Fig. 6:**
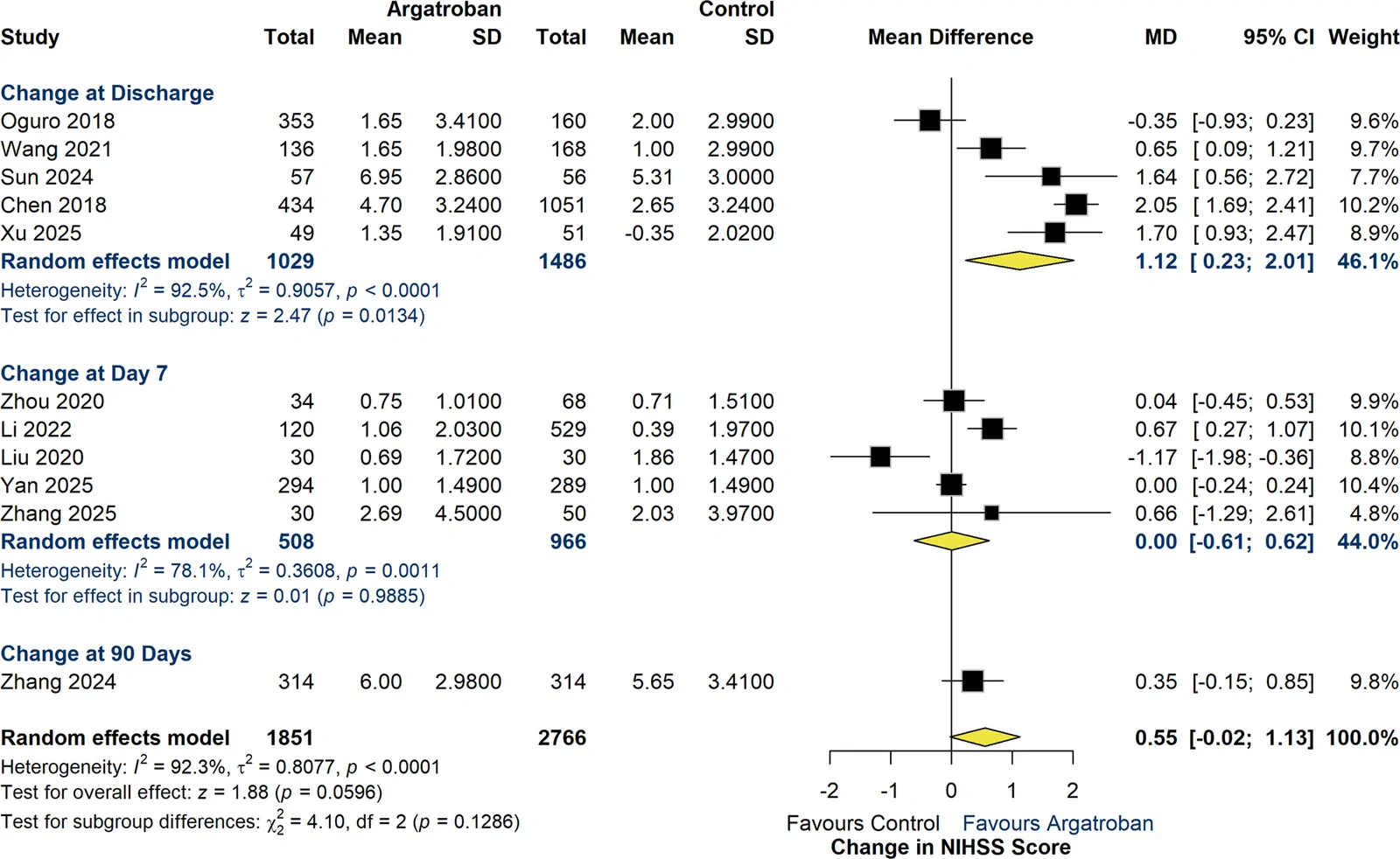
Forest plot showing the MD of change in NIHSS score for argatroban vs. control

Argatroban with antiplatelet therapy (*n* = 1,851) was not associated with a statistically significant overall improvement in NIHSS score change compared with control (*n* = 2,766), with a pooled MD of 0.55 (95% CI: − 0.02 - 1.13, *p* = 0.059, I² = 92.3%; Fig. 6). The results of the subgroup analysis based on the follow-up time points showed that among the 5 studies assessing NIHSS change at hospital discharge, argatroban was associated with a statistically significant improvement (MD: 1.12, 95% CI: 0.23–2.01, *p* = 0.01). Among the 5 studies assessing change at day 7, no difference was observed (MD: 0.00, 95% CI: − 0.61 to 0.62, *p* = 0.98). A single study reporting NIHSS change at day 90 showed a non-significant trend favoring argatroban (MD: 0.35, 95% CI: − 0.15 - 0.85). The test for subgroup differences did not reach statistical significance (χ² = 4.10, df = 2, *p* = 0.12), indicating that the apparent discrepancy between the discharge and day-7 subgroups cannot be considered statistically confirmed. Leave-one-out analysis did not yield any significant reduction in heterogeneity (Supplementary Fig. 8)

##### Mortality

**Fig. 7:**
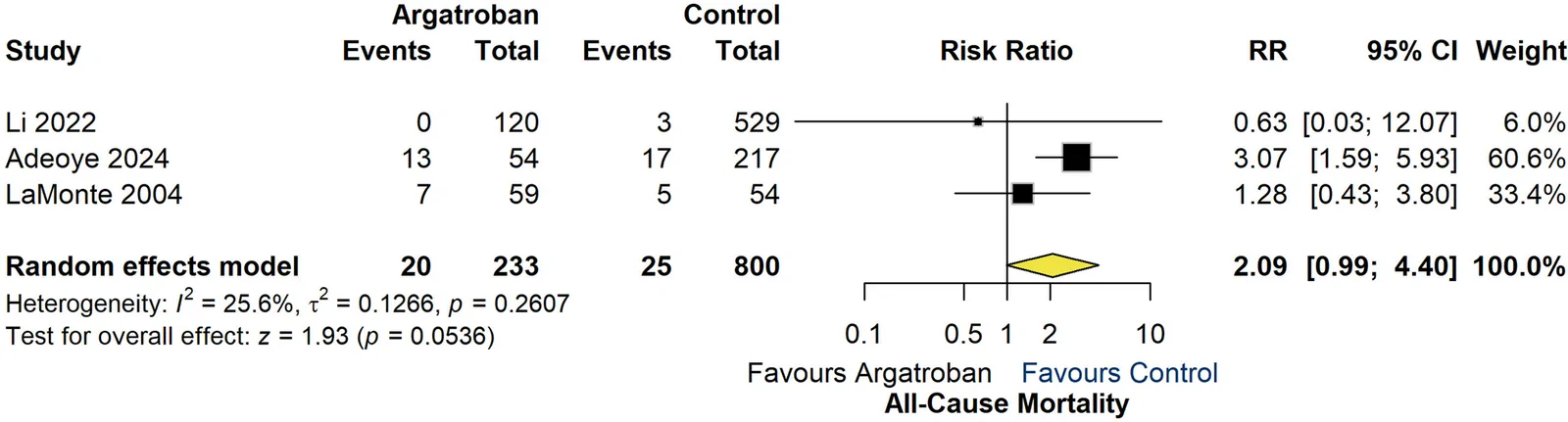
Forest plot showing the pooled RR of all-cause mortality for argatroban vs. control

The incidence of all-cause mortality was comparable between the control (*n* = 800) and argatroban group (*n* = 223), with an RR of 2.09 (95% CI: 0.99–4.40, *p* = 0.053, I^2^ = 25.6%; Fig. 7). The 7-day mortality was similarly comparable (RR: 0.84 95% CI: 0.56–1.26, *p* = 0.39, I^2^ = 0.0%; supplementary Fig. 9).

##### Intracranial Hemorrhage, Stroke Recurrence, and Organ Hemorrhage

**Fig. 8:**
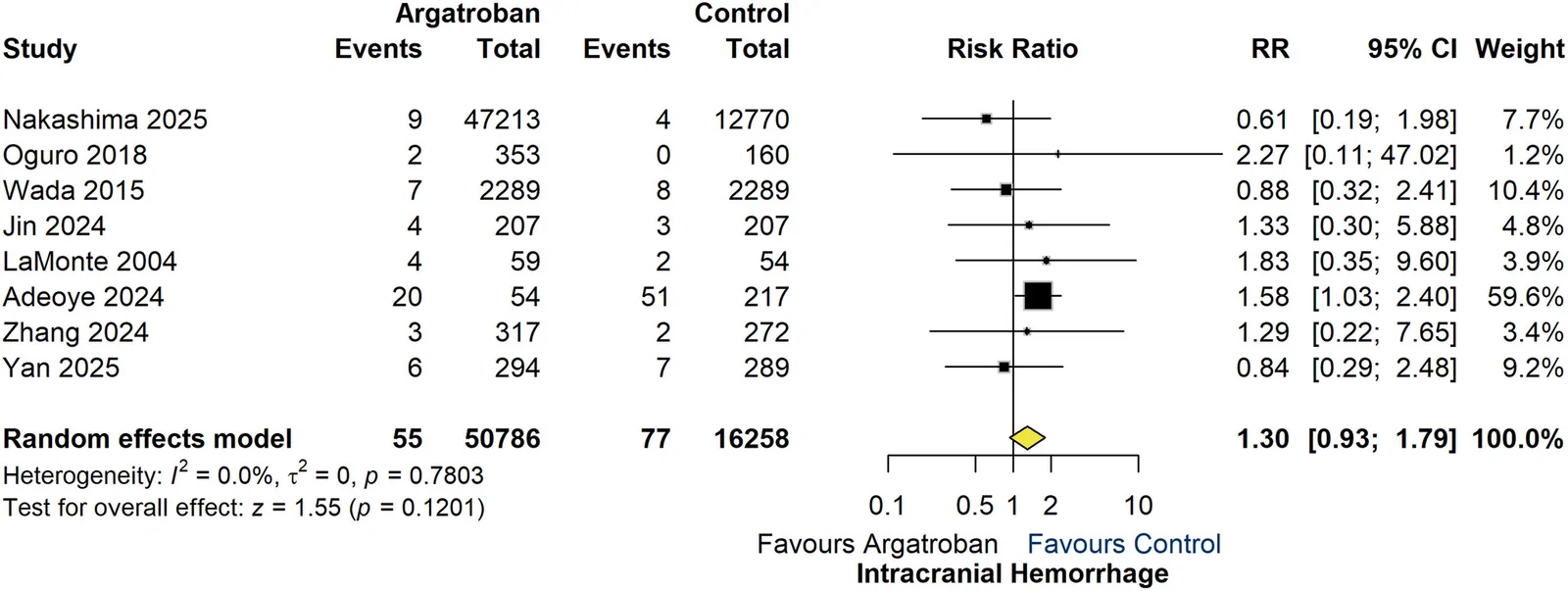
Forest plot showing the pooled RR of intracranial hemorrhage for argatroban vs. control

**Fig. 9:**
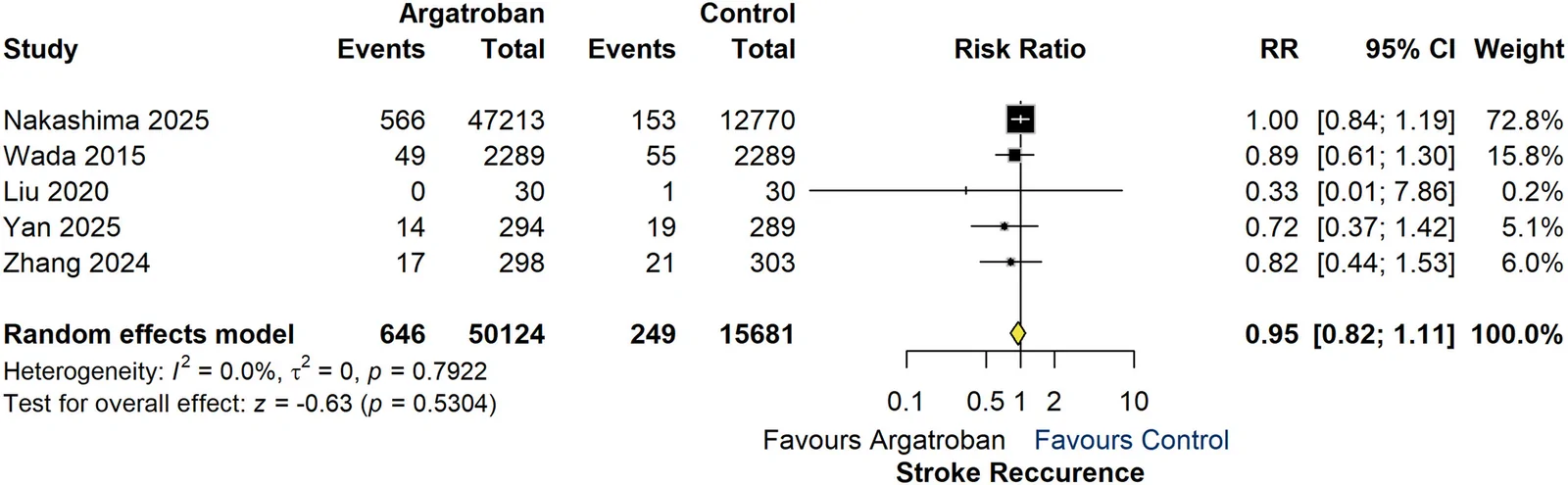
Forest plot showing the pooled RR of stroke recurrence for argatroban vs. control

**Fig. 10:**
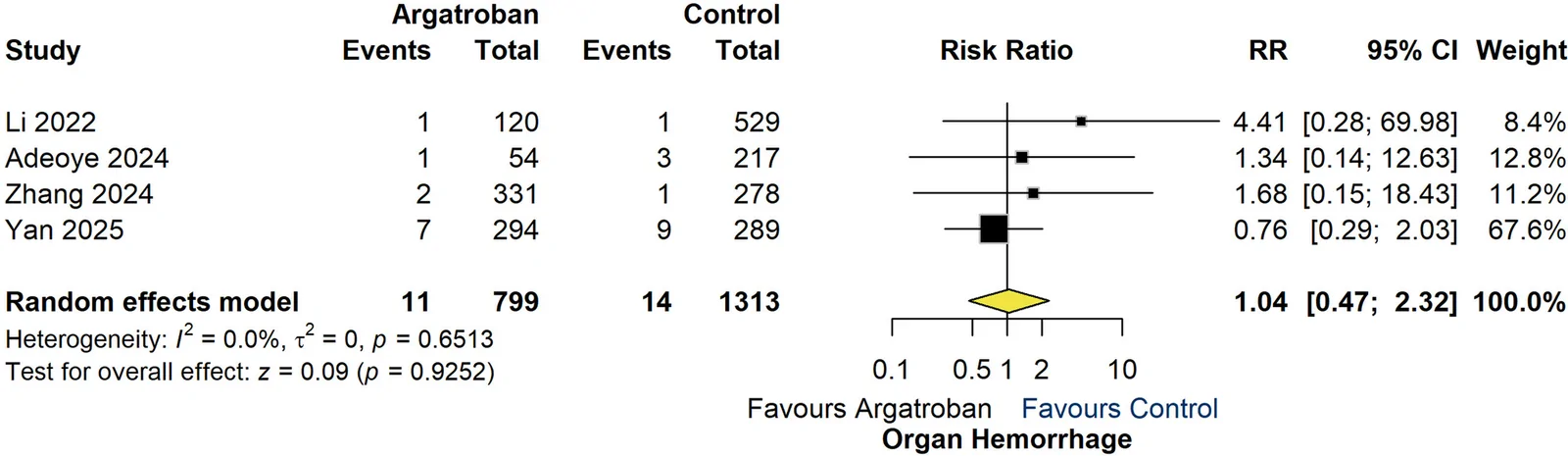
Forest plot showing the pooled RR of organ hemorrhage for argatroban vs. control

Argatroban and the control group were comparable in increasing the risk of intracranial hemorrhage and stroke recurrence with RR: 1.30 (95% CI: 0.93–1.79, *p* = 0.12) and 0.95 (95% CI: 0.82–1.11, *p* = 0.53), respectively (Figs. 8 and 9). There was also no difference in risk of organ hemorrhage with RR: 1.04 (95% CI: 0.47–2.32, *p* = 0.92; Fig. 10).

#### Subgroup analysis

The following subgroup analyses were conducted as exploratory investigations to identify potential sources of heterogeneity and generate hypotheses for future research. Given the multiple comparisons performed across intervention, comparison, study design, risk-of-bias and stroke phenotype subgroups, the risk of type I error is substantially elevated, and all subgroup findings must be interpreted as exploratory rather than confirmatory.

##### Subgroup Analysis By Comparison

**Fig. 11:**
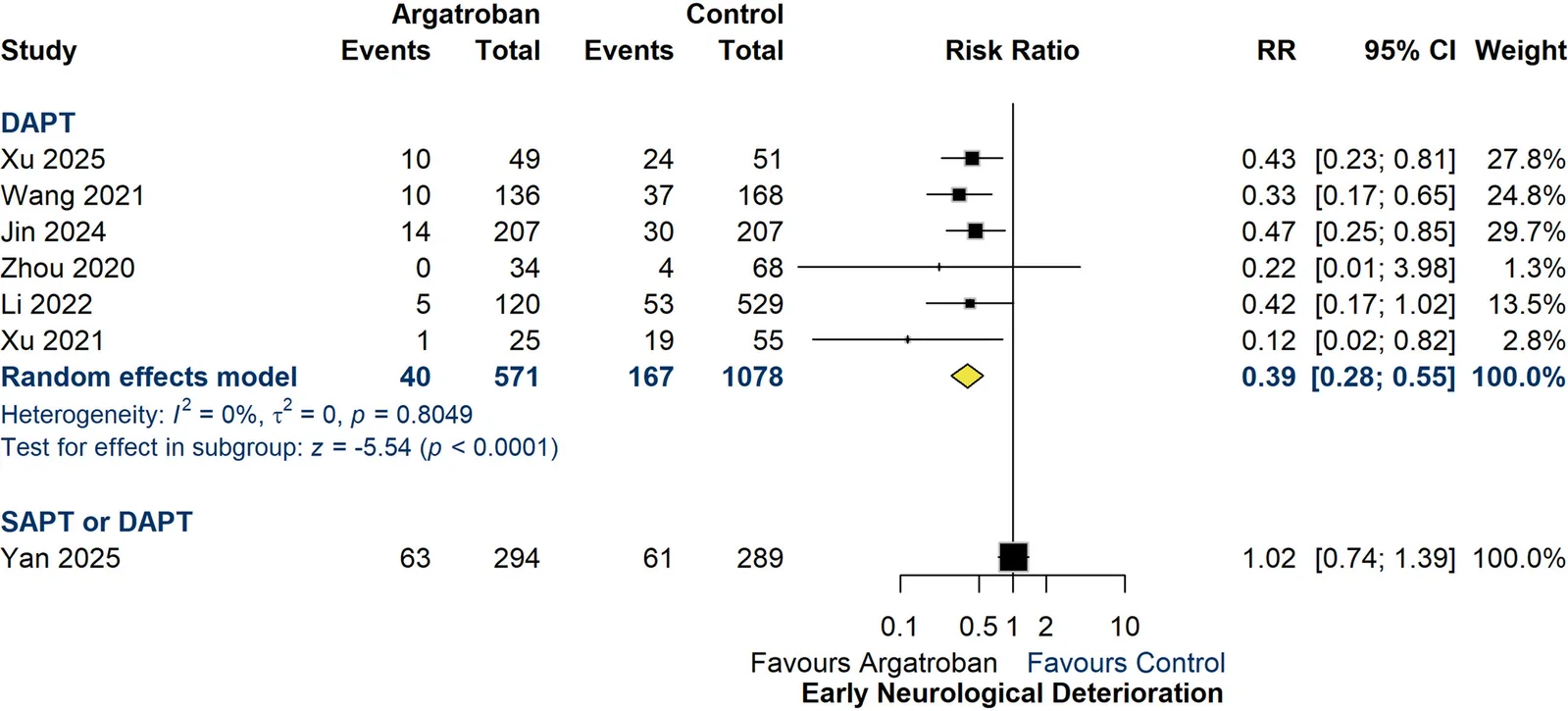
Forest plot showing the subgroup analysis of END based on the comparison group

**Fig. 12:**
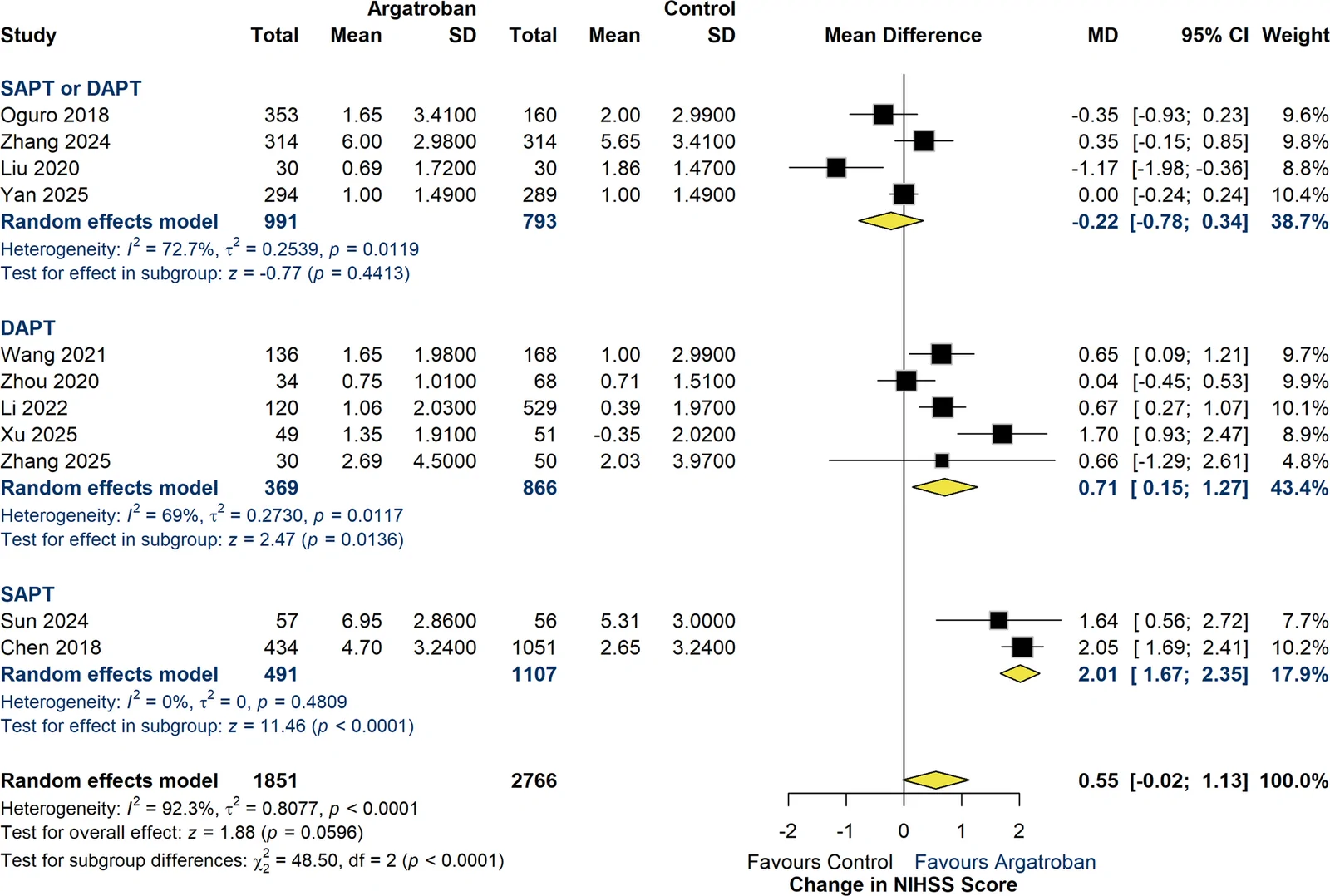
Forest plot showing the subgroup analysis of change in NIHSS score based on the comparison group

In the case of END, the DAPT subgroup showed a statistically significant risk reduction, with an RR: 0.39 (95% CI: 0.28–0.55, *p* < 0.0001). In contrast, only a single study was available for the argatroban plus SAPT or DAPT subgroup (Fig. 11). Subgroup analysis by comparison showed a significant improvement in NIHSS score for the DAPT subgroup (MD: 0.71, 95% CI: 0.15–1.27; *p* = 0.01) and SAPT subgroup (MD: 2.01, 95% CI: 1.67–2.35; *p* < 0.0001), with no difference in the SAPT or DAPT subgroup. Results are presented in Fig. 12. The DAPT subgroup showed a decrease in mRS (0–1) (*p* = 0.004; Supplementary Fig. 10)*.* Subgroup analysis for mRS (0–2), composite hemorrhage, intracranial hemorrhage, stroke recurrence and organ hemorrhage showed no statistically significant effect in any subgroup; Supplementary Figs. 11, 12, 13, 14, 15.

##### Subgroup Analysis by Intervention

**Fig. 13:**
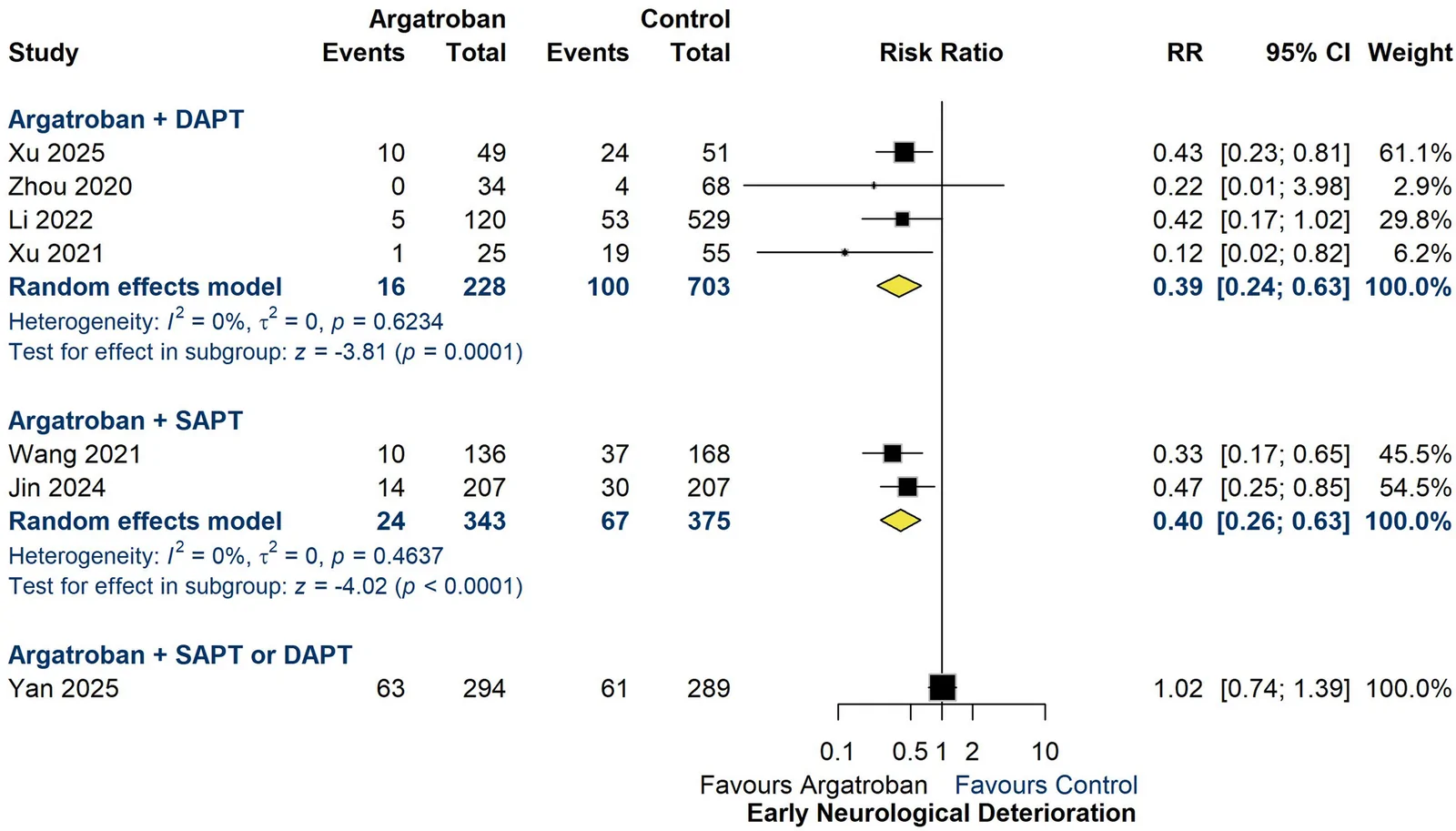
Forest plot showing the subgroup analysis of END based on the intervention group

**Fig. 14:**
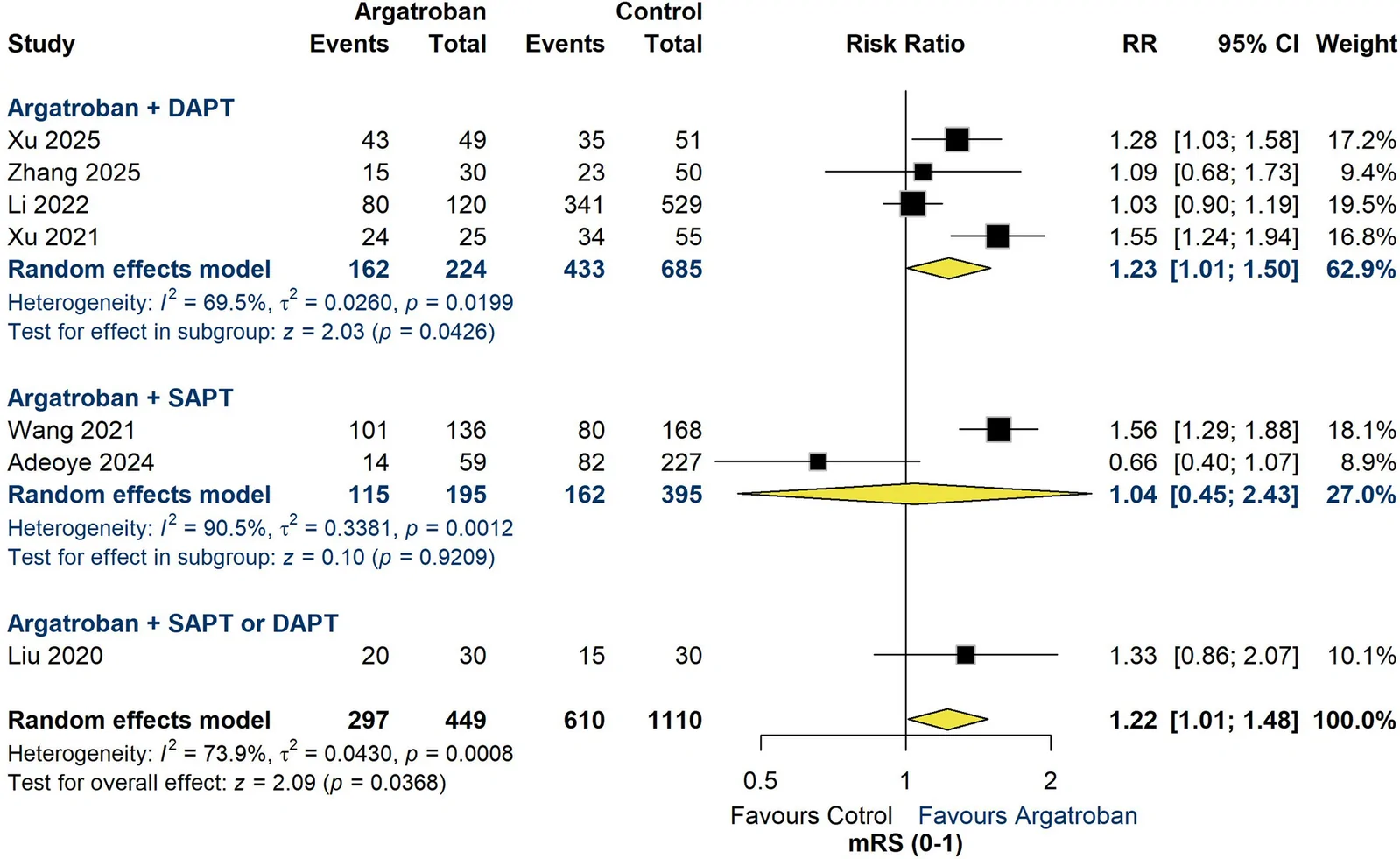
Forest plot showing the subgroup analysis of mRS (0–1) based on the intervention group

**Fig. 15:**
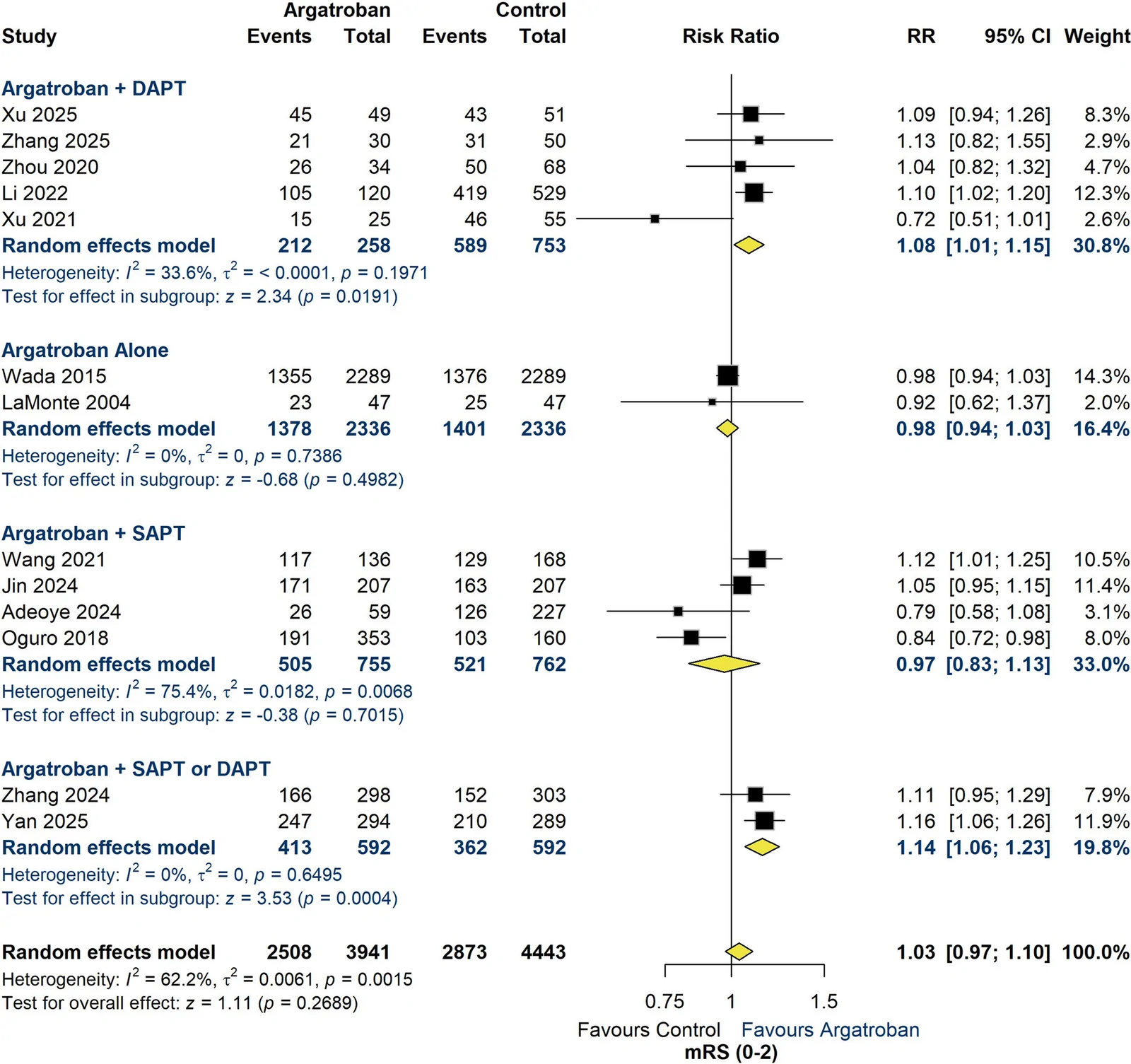
Forest plot showing the subgroup analysis of mRS (0–2) based on the intervention group

Subgroup analysis by intervention for END demonstrated that the argatroban plus DAPT subgroup significantly favoured argatroban with RR: 0.39 (95% CI: 0.24–0.63, *p* = 0.0001). The argatroban plus SAPT subgroup also showed a statistically significant benefit, with an RR of 0.40 (95% CI, 0.26–0.63; *p* < 0.0001). (Fig. 13). Similarly, subgroup analysis for mRS (0–1) revealed that argatroban plus DAPT subgroup favored better outcomes (RR: 1.23, 95% CI: 1.01–1.50; *p* = 0.04; Fig. 14). Moreover, argatroban plus DAPT (*p* = 0.01) and argatroban plus SAPT or DAPT (*p* = 0.0004) subgroups improved mRS (0–2). Results are presented in Fig. 15. Subgroup analysis for change in NIHSS score, composite hemorrhage, ICH, stroke recurrence and organ hemorrhage did not yield statistically significant findings in any subgroup (Supplementary Figs. 16, 17, 18, 19, 20).

##### Subgroup Analysis by Study Design

Subgroup analysis by study design for mRS (0–2) and composite hemorrhage revealed that RCTs demonstrated a statistically significant benefit, favoring argatroban with an RR of 1.08 (95% CI: 1.02–1.15; *p* = 0.01) and 2.17 (95% CI: 1.07–4.41; *p* = 0.03), respectively. For mRS (0–1), retrospective cohort studies significantly favored argatroban (RR: 1.51, 95% CI: 1.32–1.73; *p* < 0.0001), while RCTs did not reach statistical significance (RR:1.08, 95% CI: 0.86–1.35; *p* = 0.49). Subgroup analysis by study design for END showed that the RCTs statistically significantly favored argatroban with an RR of 0.43 (95% CI: 0.26–0.71; *p* = 0.001). While the same was true for the retrospective cohort group as well, which had an RR of 0.47 (95% CI: 0.24–0.92; *p* = 0.02). For intracranial hemorrhage specifically, RCTs again showed a significant increase in events with argatroban (RR 1.57, 95% CI: 1.06–2.35; *p* = 0.02), while retrospective cohort studies showed no such signal (RR 0.88, 95% CI: 0.50–1.54; *p* = 0.64). Details of these findings are presented in the Supplementary Table 3*.*

##### Subgroup Analysis by Risk of Bias Grading

Subgroup analysis by study quality assessment composite hemorrhage showed that the low-risk subgroup was the only one to reach statistical significance, showing a significantly higher rate of hemorrhagic events with argatroban (RR: 2.17, 95% CI: 1.07–4.41; *p* = 0.03), while high-risk (RR: 1.11, 95% CI: 0.97–1.26; *p* = 0.12) and moderate-risk (RR: 0.93, 95% CI: 0.70–1.25; *p* = 0.64) subgroups were non-significant. The moderate-risk subgroup for change in NIHSS score was the only one to demonstrate a statistically significant reduction favoring argatroban (MD: 0.46, 95% CI: 0.05–0.86; *p* = 0.02), whereas neither the high-risk (MD: 0.79, 95% CI: −0.22–1.79; *p* = 0.12) nor the low-risk subgroup (MD: 0.30, 95% CI: −1.30–1.90; *p* = 0.71) reached significance. For END, the moderate-risk subgroup significantly favored argatroban (RR: 0.36, 95% CI: 0.21–0.60; *p* = 0.0001), as did the single low-risk study (Xu 2025; RR: 0.43, 95% CI: 0.23–0.81), while the high-risk subgroup did not reach significance (RR: 0.53, 95% CI: 0.21–1.37; *p* = 0.19). For intracranial hemorrhage, the low-risk subgroup showed a statistically significant increase in events with argatroban (RR: 1.57, 95% CI: 1.06–2.35; *p* = 0.02). Details of these results are provided in the Supplementary Table 4.

##### Subgroup Analysis by Stroke Phenotype

Subgroup analysis by stroke phenotype for mRS (0–2) and composite hemorrhage did not reveal any statistical significant effect in any subgroup. For mRS (0–1), only the small vessel/BAD spectrum subgroup statistically significantly favored argatroban with RR of 1.43, 95% CI: 1.26–1.63; *p* < 0.0001. Similarly, for END and NIHSS score change, only the small vessel/BAD spectrum subgroup favored argatroban significantly (RR: 0.36, 95% CI: 0.23–0.56; *p* < 0.0001 and MD: 1.06, 95% CI: 0.25–1.88; *p* = 0.01; respectively). Details of these results are provided in the Supplementary Table 5.

##### Certainty of Evidence

The certainty of evidence for all primary and secondary outcomes was assessed using the GRADE framework restricted to the six RCTs included in the RCT-only subgroup analysis. This was done to provide the most methodologically rigorous certainty estimate unconfounded by limitations of observational studies. The certainty of evidence for the primary outcomes, mRS (0–2) and composite hemorrhage, was rated as moderate and low, respectively. It was downgraded for mRS (0–2) due to serious risk of bias, while serious indirectness and imprecision were the reasons for composite hemorrhage. For mRS (0–1), END and change in NIHSS score, the certainty is very low, moderate and low, respectively. Intracranial hemorrhage and stroke recurrence had high and moderate certainty of evidence, respectively. The results of the assessment have been provided in Supplementary Table 6*.*

## Discussion

The results of our updated systematic review and meta-analysis suggest that although the primary outcomes mRS (0–2) and composite hemorrhage did not reach statistical significance, argatroban demonstrated consistent and significant benefits across secondary neurological outcome, END. The variability in the type of antiplatelet therapy used along with argatroban in the intervention group and the antiplatelet drugs used in the control group contributed to potential heterogeneity in the results. This was addressed by performing subgroup analyses based on the differing intervention and control groups, study design, risk of bias assessment and stroke phenotype. The results of our exploratory subgroup analyses indicated a significant reduction in END when argatroban was combined with DAPT alone.

Our analysis revealed no significant improvement in the overall functional outcome mRS (0–2) with argatroban along with antiplatelet therapy compared with the control. These findings align closely with Cheng et al. [41], who reported no significant improvements in the functional outcomes in the argatroban group, suggesting that while argatroban can have a major role in acute stabilization of neurological symptoms, its benefit in improving the long-term functional and disability outcomes is limited [42]. However, a recent meta-analysis by Ahmed et al. [12] reported a statistically significant improvement in mRS (0–1) and mRS (0–2) in patients receiving argatroban therapy. We found that the argatroban group was 22% more likely to achieve a functional score of mRS (0–1). Scores of 0–1 are generally considered an excellent functional outcome, while a score of 2 represents slight disability where the patient cannot carry out all previous activities, even if still independent in self-care [43]. The fact that argatroban shows a significant benefit at this stricter threshold indicates that the drug is moving patients from slight disability into complete or near-complete neurological recovery, a meaningfully higher bar with direct implications for patients’ quality of life and return to pre-morbid function. However, these results should be interpreted alongside the finding of our subgroup analysis based on study design. This analysis revealed that the mRS (0–1) benefit was driven entirely by observational data and was not replicated within the RCT subgroup.

In our study, there was no significant increase in the risk of composite hemorrhage in the argatroban group as compared to the control, emphasizing that the anticoagulant effect of argatroban does not necessarily lead to a higher burden of bleeding in the setting of ischemic stroke. This is particularly important as the use of anticoagulants in the early management of ischemic stroke often raises concerns for an increased chance of bleeding [42]. Previous studies have reported that the use of argatroban in patients with AIS does not increase the risk of bleeding [44, 45]. Nonetheless, the bleeding risk of the patient should be promptly assessed, and other factors such as atherosclerosis should be taken into account, as for those with lower bleeding risk, the combination of argatroban and antiplatelets can prove to be beneficial [44].

However, this overall safety conclusion must be interpreted with important caveats. Subgroup analysis stratified by study design revealed a markedly different safety profile when evidence was restricted to RCTs. Within the RCT subgroup, argatroban was associated with a statistically significant increase in composite and intracranial hemorrhage. This discrepancy between the overall pooled estimate and the RCT-specific signal is clinically important. Observational studies, which comprise the majority of included studies and were predominantly rated as serious risk of bias on ROBINS-I assessment, may systematically underestimate hemorrhagic complications due to selection bias and retrospective outcome ascertainment. The RCT-derived hemorrhage signal therefore represents a higher-quality and more reliable estimate of the true bleeding risk associated with argatroban, and should be prominently considered in clinical decision-making. These findings point that the balance between the neurological benefits and hemorrhagic risks of argatroban requires careful individualized assessment, with particular attention to patient-level bleeding risk factors prior to initiating therapy.

Our findings support argatroban regarding the improvement in early neurological outcomes in patients with AIS. This effect has been most consistently reflected in the reduction of END across subgroup analyses, particularly when argatroban was combined with DAPT. The DAPT-specific subgroup finding, while biologically plausible, was derived from an exploratory analysis and is subject to the well-documented risk of spurious significance in multiple-comparison settings. Rothwell et al. have demonstrated that subgroup analyses in clinical trials and meta-analyses are prone to false-positive findings, particularly when not prespecified [46].

In contrast to END, our analysis of change in NIHSS score change revealed a less consistent picture. The overall pooled effect did not reach statistical significance; however, subgroup analysis by assessment timepoint showed a significant improvement among studies reporting NIHSS change at hospital discharge, but no effect among studies assessed at day 7. Cheng et al. [40] and Chen et al. [30] similarly reported reductions in NIHSS score with argatroban, though direct comparison is limited by differences in the specific assessment timepoints used across studies. Any NIHSS-modifying effect of argatroban appears to be time-dependent and should be regarded as hypothesis-generating. The more robust and consistent finding across our analyses remains the reduction in END, which may reflect argatroban’s effects on microvasculature, including inhibition of platelet aggregation and improved cerebral blood flow [26].

Our results about the comparability of argatroban and the control group in reducing 7-day and all-cause mortality are consistent with findings of previous studies [14, 27, 31]. Similarly, previous studies that assessed the efficacy and safety of argatroban therapy in patients with Heparin Induced Thrombocytopenia (HIT) found no significant reductions in all-cause and 7-day mortality [47, 48].

Our meta-analysis differs from the previous meta-analyses [12, 41, 42] on argatroban for ischemic stroke in several different ways. First, our meta-analysis encompasses a larger and more recent pool of studies, with a total of 17 studies being incorporated, including three new studies and a comprehensive review of the previously existing literature. The cumulative patient population in this review, 51,703 in the argatroban group and 18,759 in the control group, substantially exceeds that of all prior meta-analyses in this area. Such a sample size enhances the precision of pooled estimates. Secondly, this review presents a more robust methodology and detailed analysis by categorization of different antiplatelet regimens (DAPT, SAPT, and DAPT or SAPT). We performed subgroup analyses based on the antiplatelet therapy used in the intervention and control groups, study design, risk of bias assessment and stroke phenotype. Third, we have further strengthened our findings by performing a GRADE assessment of the RCT-only subgroup. Fourth, our review has taken into account a broad range of clinical outcomes, including mRS (0–1), mRS (0–2), NIHSS, END, composite hemorrhage, recurrence of stroke, and mortality. These findings can potentially improve the clinical applicability of the use of argatroban as an adjunct to antiplatelet therapy.

There are a few limitations of our study. First, substantial statistical heterogeneity has been observed across several pooled outcomes. Five consequential reasons for this heterogeneity are worth mentioning. The inclusion criteria, which combined both RCTs and observational studies which are more prone to confounding and selection bias, resulted in heterogeneity. The included studies enrolled patients across a broad spectrum of stroke phenotypes, including minor ischemic stroke, lacunar infarction, branch atherosclerotic disease, posterior circulation infarction, large artery atherosclerosis, and post-thrombolysis settings. These subtypes differ fundamentally in their pathophysiology, natural history, and responsiveness to antithrombotic intensification. Similarly, there was an inconsistency in the definition of END, in terms of the magnitude of NIHSS change considered deteriorating (ranging from ≥ 2 to ≥ 4 points), the timeframe within which deterioration was assessed (ranging from 24 h to 7 days after onset), and the reference point used (admission NIHSS vs. symptom-onset NIHSS). This added methodological heterogeneity to the END outcome. The varying definitions of END as reported by different studies are presented in Supplementary Table 7. Although a formal subgroup analysis stratified by END definition was not feasible due to insufficient numbers of studies within each definitional subgroup, future studies should adopt a standardized END definition to enable more reliable pooling and interpretation of this outcome. Furthermore, the timing of argatroban initiation varied from within 6 h to 72 h after symptom onset across included studies. The therapeutic window for antithrombotic therapy in AIS is time-sensitive. Studies initiating argatroban within the hyperacute phase may capture a meaningfully different treatment effect from those initiating it at 48–72 h, when the acute thrombotic process may have already evolved. This variability may attenuate the pooled END and NIHSS estimates by diluting the early treatment effect across studies with delayed initiation. The concomitant antiplatelet regimen varied substantially, ranging from aspirin monotherapy to dual antiplatelet therapy with varying loading doses, introducing pharmacodynamic heterogeneity in the degree of platelet inhibition achieved on top of argatroban. The subgroup analyses by antiplatelet regimen were performed specifically to address this dimension, and the consistently stronger effects observed in the DAPT subgroup suggest that antiplatelet co-administration is an important effect modifier. Together, these five sources of heterogeneity must be considered when interpreting the pooled estimates, which represent a broad average treatment effect across a clinically diverse population rather than a subtype-specific or protocol-specific treatment effect. However, the random-effects model used throughout this analysis was selected specifically to accommodate the anticipated between-study clinical variability.

Second, the conduction of multiple subgroup analyses increases the risk of type I error inflation, whereby one or more subgroup findings may represent false positives arising from chance rather than true differential treatment effects. Subgroup findings should therefore be interpreted with caution and regarded as exploratory rather than definitive. Prospective RCTs with pre-registered subgroup analyses stratified by antiplatelet regimen represent the necessary next step for confirmatory testing of these subgroup results., Third, retrospective cohort studies are predominant in this systematic review and meta-analysis. The majority of these studies were judged to have a serious risk of bias on assessment. Retrospective designs are inherently susceptible to selection bias and confounding by indication. That is why we conducted a subgroup analysis stratifying the studies based on their risk of bias assessment. The results of our analysis showed that, RCT-only subgroup favored argatroban for mRS (0–2) and END. However, the risk of composite and intracranial hemorrhage was also elevated in this subgroup. The single outcome that did not reach significance under randomized evidence was mRS (0–1), which was significant only in the observational subgroup. At the same time, the inclusion of real-world cohort studies, while a source of methodological heterogeneity, enhances the generalizability of findings to everyday clinical practice, where patient populations are less selected and treatment conditions are less controlled than in randomized trial settings. Fourth, the Hartung–Knapp–Sidik–Jonkman (HKSJ) adjustment was not applied in the primary analysis. While HKSJ can provide more conservative and appropriately widened confidence intervals in meta-analyses with few studies, it was not implemented here in order to maintain consistency with the analytic approach of the original review being updated. Also, our primary conclusions are based on the overall pattern of effect sizes and their consistency across sensitivity and subgroup analyses rather than on borderline p-values for any single outcome. Fifth, the majority of the studies included in this review had a small sample size, which reduced the statistical power and generalizability of the results. Hence, larger RCTs and cohorts are required to further investigate the efficacy and safety of argatroban as an add-on to antiplatelet therapy.

## Conclusion

Argatroban, when used along with antiplatelet therapy in patients with AIS, did not demonstrate a statistically significant improvement in mRS (0–2) or reduction in composite hemorrhage. However, it was associated with a significant reduction in END and modest improvement in NIHSS scores, suggesting a potential role in short-term neurological stabilization. It is noteworthy that the statistical significance of change in NIHSS score at discharge has limited clinical significance. Given that the predefined primary outcome did not reach statistical significance, these secondary findings must be regarded as hypothesis-generating rather than confirmatory evidence of efficacy. They should be interpreted with caution in light of the predominantly observational design of the included studies, the serious risk of bias of included studies and the persistent heterogeneity observed across analyses. The efficacy of argatroban in improving these outcomes is most pronounced when it is used with DAPT alone. But this interaction requires prospective testing before informing treatment selection in clinical practice. Although the overall pooled analysis did not demonstrate a statistically significant increase in composite hemorrhage or mortality, subgroup analysis restricted to RCTs revealed a significant increase in both composite and intracranial hemorrhage with argatroban. Given that RCT-derived evidence carries substantially higher methodological credibility than retrospective observational data, this safety signal cannot be dismissed and must be carefully considered when weighing the potential neurological benefits of argatroban against its bleeding risks in clinical practice. Nevertheless, the overall very low to high certainty of evidence on GRADE assessment depicts the methodological limitations of the included RCT evidence.

## Supplementary Information

Below is the link to the electronic supplementary material.

Supplementary File 1 (PDF 267 KB)

Supplementary File 2 (DOCX 32.9 KB)

Supplementary File 3 (PDF 64.9 KB)

Supplementary File 4 (DOCX 268 KB)

Supplementary File 5 (DOCX 7.83 MB)

## Data Availability

No datasets were generated or analysed during the current study.

## References

[1] Siegler JE, Martin-Schild S (2011) Early neurological deterioration (END) after stroke: the end depends on the definition. Int J Stroke 6(3):211–212. 10.1111/j.1747-4949.2011.00596.x 21557807

[2] Huang ZX, Wang QZ, Dai YY, Lu HK, Liang XY, Hu H et al (2018) Early neurological deterioration in acute ischemic stroke: A propensity score analysis. J Chin Med Assoc 81(10):865–870. 10.1016/j.jcma.2018.03.011 30093284

[3] Wang Y, Wang Y, Zhao X, Liu L, Wang D, Wang C et al (2013) Clopidogrel with aspirin in acute minor stroke or transient ischemic attack. N Engl J Med 369(1):11–19. 10.1056/NEJMoa1215340 23803136

[4] Khatri P, Conaway MR, Johnston KC (2012) Ninety-day outcome rates of a prospective cohort of consecutive patients with mild ischemic stroke. Stroke 43(2):560–2. 10.1161/STROKEAHA.110.593897 PMC3426999 22052513

[5] Johnston SC, Amarenco P, Denison H, Evans SR, Himmelmann A, James S et al (2020) Ticagrelor and aspirin or aspirin alone in acute ischemic stroke or TIA. N Engl J Med 383(3):207–17. 10.1056/NEJMoa1916870 32668111

[6] Pan Y, Jing J, Chen W, Meng X, Li H, Zhao X et al (2017) Risks and benefits of clopidogrel-aspirin in minor stroke or TIA: time course analysis of CHANCE. Neurology 88(20):1906–11. 10.1212/WNL.0000000000003941 28424269

[7] Toyoda K, Uchiyama S, Yamaguchi T, Easton JD, Kimura K, Hoshino H et al (2019) Dual antiplatelet therapy using cilostazol for secondary prevention in patients with high-risk ischaemic stroke in Japan: a multicentre, open-label, randomised controlled trial. Lancet Neurol 18(6):539–48. 10.1016/S1474-4422(19)30148-6 31122494

[8] Cui C, Li C, Hou M, Wang P, Huang Z (2023) The machine learning methods to analyze the using strategy of antiplatelet drugs in ischaemic stroke patients with gastrointestinal haemorrhage. BMC Neurol 23:369. 10.1186/s12883-023-03422-0 PMC10571309 37833629

[9] Jin X, Li X, Zhang H, Yao X, Gu Y, Pei S et al (2024) The effect of argatroban on early neurological deterioration and outcomes in minor ischemic stroke: preliminary findings. Front Neurol 15:1363358. 10.3389/fneur.2024.1363358 PMC10957773 38523614

[10] Desai J, Kolb J, Weitz J, Aisenberg J (2013) Gastrointestinal bleeding with the new oral anticoagulants – defining the issues and the management strategies. Thromb Haemost 110(08):205–212. 10.1160/TH13-02-0150 23702623

[11] Wang PF, Sun ZR, Yu JC, Geng N, Liu LY, Zhu LN et al (2021) Early argatroban and antiplatelet combination therapy in acute non-lacunar single subcortical infarct associated with mild intracranial atherosclerosis. BMC Neurol 21:440. 10.1186/s12883-021-02435-x PubMed PMID: 34753435; PubMed Central PMCID: PMC8579679 PMC8579679 34753435

[12] Ahmed Y, Moawad MHED, Bahtiyarova G, Nabgouri Y, Elkholy M, Wagih RM et al (2025) Exploring the efficacy and safety of argatroban as an adjunct to antiplatelet therapy in ischemic stroke patients: a systematic review and meta-analysis. Transl Stroke Res. 10.1007/s12975-025-01357-x PMC12596288 40392513

[13] Xu J, Liu Y, Wang H, Sun R, Zhao H, Liu X et al (2025) Effect of argatroban plus dual antiplatelet in branch atherosclerosis disease: a randomized clinical trial. Stroke. 10.1161/STROKEAHA.124.048872 PMC12180705 40242875

[14] Nakashima S, Kodama S, Aso S, Jo T, Yasunaga H, Isogai T et al (2025) Argatroban plus dual antiplatelet therapy versus dual antiplatelet alone for acute atherothrombotic cerebral infarction. Int J Stroke. 10.1177/17474930251344458 40375365

[15] Page MJ, McKenzie JE, Bossuyt PM, Boutron I, Hoffmann TC, Mulrow CD et al (2020) The PRISMA 2020 statement: an updated guideline for reporting systematic reviews. BMJ. 10.1136/bmj.n71 PMC8005924 33782057

[16] Shea BJ, Reeves BC, Wells G, Thuku M, Hamel C, Moran J et al (2017) AMSTAR 2: a critical appraisal tool for systematic reviews that include randomised or non-randomised studies of healthcare interventions, or both. BMJ. 10.1136/bmj.j4008 PMC5833365 28935701

[17] Ouzzani M, Hammady H, Fedorowicz Z, Elmagarmid A (2016) Rayyan—a web and mobile app for systematic reviews. Syst Rev 5(1):210. 10.1186/s13643-016-0384-4 PMC5139140 27919275

[18] Broderick JP, Adeoye O, Elm J (2017) The evolution of the modified rankin scale and its use in future stroke trials. Stroke 48(7):2007. 10.1161/STROKEAHA.117 PMC5552200 28626052

[19] Suryanarayana Sharma PM, Tekkatte Jagannatha A, Javali M, Hegde AV, Mahale R, Madhusudhan et al (2015) Spontaneous subdural hematoma and antiplatelet therapy: Does efficacy of Ticagrelor come with added risk? Indian Heart J 67(Suppl 3):S30–S35 .024 PubMed PMID: 26995425; PubMed Central PMCID: PMC4798975 10.1016/j.ihj.2015.06.024 PMC4798975 26995425

[20] NIH Stroke Scale | National Institute of Neurological Disorders and Stroke [Internet]. [cited 2026 Feb 24]. Available from: https://www.ninds.nih.gov/health-information/stroke/assess-and-treat/nih-stroke-scale

[21] Sterne JAC, Savović J, Page MJ, Elbers RG, Blencowe NS, Boutron I et al (2019) RoB 2: a revised tool for assessing risk of bias in randomised trials. BMJ l4898. 10.1136/bmj.l4898 31462531

[22] Sterne JA, Hernán MA, Reeves BC, Savović J, Berkman ND, Viswanathan M et al (2016) ROBINS-I: a tool for assessing risk of bias in non-randomised studies of interventions. BMJ. 10.1136/bmj.i4919 PMC5062054 27733354

[23] Mantel N, Haenszel W (1959) Statistical aspects of the analysis of data from retrospective studies of disease. JNCI J Natl Cancer Inst. 10.1093/jnci/22.4.719 13655060

[24] Tanriver-Ayder E, Faes C, van de Casteele T, McCann SK, Macleod MR (2021) Comparison of commonly used methods in random effects meta-analysis: application to preclinical data in drug discovery research. BMJ Open Sci 5(1):e100074. 10.1136/bmjos-2020-100074 PMC8647574 35047696

[25] Zhang H, Ma A, Ma H, Chang W, Liu H, Chen J et al (2025) Argatroban plus dual antiplatelet therapy: preliminary evidence for managing early neurological deterioration after lacunar stroke. Clin Neurol Neurosurg 250:108786. 10.1016/j.clineuro.2025.108786 40010239

[26] Zhou LS, Li XQ, Zhou ZH, Chen HS (2020) Effect of argatroban combined with dual antiplatelet therapy on early neurological deterioration in acute minor posterior circulation ischemic stroke. Clin Appl Thromb 26:1076029620904131. 10.1177/1076029620904131 PMC7288810 32013541

[27] Li X, Hou X, Cui Y, Tian X, Wang X, Zhou Z et al (2022) Safety and preliminary efficacy of argatroban plus dual antiplatelet therapy for acute mild to moderate ischemic stroke with large artery atherosclerosis. Brain Behav 12(7):e2664. 10.1002/brb3.2664 PMC9304821 35678020

[28] Xu J, Xu X, Wang H, He L, Liu Q, Du Y et al (2022) Dual antiplatelet therapy plus argatroban prevents early neurological deterioration in branch atherosclerosis disease. Stroke. 10.1161/STROKEAHA.121.036356 34784740

[29] Sun M, Li G, Du Y, Cheng J, Zhu Q, Shi Z (2025) Efficacy of antiplatelet drugs combined with Argatroban in treating acute ischemic stroke and its impact on patients’ coagulation function and neurological function: a preliminary trial. Int J Neurosci 135(4):405–412. 10.1080/00207454.2024.2303370 38193210

[30] Chen L, Cao S, Yang J (2018) Argatroban plus aspirin versus aspirin in acute ischemic stroke. Neurol Res 40(10):862–867. 10.1080/01616412.2018.1495882 30052164

[31] LaMonte MP, Nash ML, Wang DZ, Woolfenden AR, Schultz J, Hursting MJ et al (2004) Argatroban anticoagulation in patients with acute ischemic stroke (ARGIS-1): a randomized, placebo-controlled safety study. Stroke 35(7):1677–1682. 10.1161/01.STR.0000131549.20581.ba 15155959

[32] Wada T, Yasunaga H, Horiguchi H, Matsubara T, Fushimi K, Nakajima S et al (2016) Outcomes of argatroban treatment in patients with atherothrombotic stroke: observational nationwide study in Japan. Stroke 47(2):471–476. 10.1161/STROKEAHA.115.011250 26670085

[33] Oguro H, Mitaki S, Takayoshi H, Abe S, Onoda K, Yamaguchi S (2018) Retrospective analysis of argatroban in 353 patients with acute noncardioembolic stroke. J Stroke Cerebrovasc Dis 27(8):2175–2181. 10.1016/j.jstrokecerebrovasdis.2018.03.016 29706441

[34] Adeoye O, Broderick J, Derdeyn CP, Grotta JC, Barsan W, Bentho O et al (2024) Adjunctive intravenous argatroban or eptifibatide for ischemic stroke. N Engl J Med 391(9):810–20. 10.1056/NEJMoa2314779 PMC11528349 39231343

[35] Yan J, Yu S, Feng H, Zhao H, Dong X, Xu Q et al (2025) Safety and efficacy of argatroban combined with antiplatelet therapy for acute mild-to-moderate ischemic stroke with large artery atherosclerosis. J Stroke Cerebrovasc Dis 34(1):108151. 10.1016/j.jstrokecerebrovasdis.2024.108151 39613198

[36] Zhang X, Zhong W, Xue R, Jin H, Gong X, Huang Y et al (2024) Argatroban in patients with acute ischemic stroke with early neurological deterioration: a randomized clinical trial. JAMA Neurol 81(2):118. 10.1001/jamaneurol.2023.5093 PMC10775075 38190136

[37] Liu S, Liu P, Wang P, Zhang F, Wang L, Wang Y et al (2020) Argatroban increased the basal vein drainage and improved outcomes in acute paraventricular ischemic stroke patients. Med Sci Monit. 10.12659/MSM.924593 PMC7382300 32667287

[38] Egger M, Smith GD, Schneider M, Minder C (1997) Bias in meta-analysis detected by a simple, graphical test. BMJ 315(7109):629–634. 10.1136/bmj.315.7109.629 PMC2127453 9310563

[39] Duval S, Tweedie R (2000) Trim and fill: a simple funnel-plot-based method of testing and adjusting for publication bias in meta-analysis. Biometrics 56(2):455–63. 10.1111/j.0006-341x.2000.00455.x 10877304

[40] Prasad M (2024) Introduction to the GRADE tool for rating certainty in evidence and recommendations. Clin Epidemiol Glob Health 25:101484. 10.1016/j.cegh.2023.101484

[41] Cheng Y, Liu C, Li S, Meng MM, Li H (2024) Efficacy and safety of argatroban in patients with acute ischemic stroke: a systematic review and meta-analysis. Front Neurol 15:1364895. 10.3389/fneur.2024.1364895 PMC10909846 38440113

[42] Lv B, Guo FF, Lin JC, Jing F (2022) Efficacy and safety of argatroban in treatment of acute ischemic stroke: A meta-analysis. World J Clin Cases 10(2):585–593. 10.12998/wjcc.v10.i2.585 PMC8771382 35097084

[43] Weisscher N, Vermeulen M, Roos YB, de Haan RJ (2008) What should be defined as good outcome in stroke trials; a modified Rankin score of 0–1 or 0–2? J Neurol 255(6):867–74. 10.1007/s00415-008-0796-8 18338195

[44] Nagakane Y, Tanaka E, Ashida S, Kojima Y, Ogura S, Maezono K et al (2018) [Safety of dual antiplatelet therapy with argatroban in patients with acute ischemic stroke]. Brain Nerve Shinkei Kenkyu No Shinpo 70(5):557–562. 10.11477/mf.1416201038 PubMed PMID: 29760293 29760293

[45] LaMonte MP, Brown PM, Hursting MJ (2004) Stroke in patients with heparin-induced thrombocytopenia and the effect of argatroban therapy. Crit Care Med 32(4):976–980. 10.1097/01.CCM.0000119426.34340.E2 15071388

[46] Rothwell PM (2005) Treating individuals 2. Subgroup analysis in randomised controlled trials: importance, indications, and interpretation. Lancet 365(9454):176–186. 10.1016/S0140-6736(05)17709-5 15639301

[47] Lewis BE, Wallis DE, Berkowitz SD, Matthai WH, Fareed J, Walenga JM et al (2001) Argatroban anticoagulant therapy in patients with heparin-induced thrombocytopenia. Circulation. 10.1161/01.CIR.103.14.1838 11294800

[48] Lewis BE, Wallis DE, Leya F, Hursting MJ, Kelton JG (2003) for the argatroban-915 investigators. argatroban anticoagulation in patients with heparin-induced thrombocytopenia. Arch Intern Med 163(15):1849–1856. 10.1001/archinte.163.15.1849 12912723

