# Supplementary material for: Efficacy and Safety of Argatroban as an Adjunct to Antiplatelet Therapy in Ischemic Stroke: An Updated Systematic Review and Meta-analysis
Source: Eur J Clin Pharmacol. 2026 Oct 22;82(11):275. doi: 10.1007/s00228-026-04203-7 (PMC13690853; doi:10.1007/s00228-026-04203-7)
Supplement: Supplementary file 3 [file 228_2026_4203_MOESM2_ESM.docx]

**Review articles**

Review articles on topics within the scope of the European Journal of Clinical Pharmacology are welcome. Both invited and unsolicited submissions may be published. Any submitted review will be peer-reviewed just as any other submission. A word limit is not specified for reviews. The Journal welcomes “full-sized” reviews of up to 4,000 words (main text) as well as “condensed” reviews or “occasional updates” of around 1,000 words. If a meta-analysis is part of a review article, please also refer to the section on “Reporting on Meta-Analyses”.

For unsolicited review articles, the following “Review article submission form” should be filled in. Respective articles not accompanied by this form with entries in all sections will be rejected without further processing.

**Review article submission form**

| 1 | ☐ Tick if this review is a **systematic** review. Else, go to section 2. |
| --- | --- |
| 2 | If it is a narrative review, explain why this procedure was favored. |
| 3 | List the most recent and/or most similar review articles published previously in the same area. |
| 4 | Explain the additional value provided by the present review as compared to the previous papers mentioned in section 3 and explain why this is important enough to justify publication of another review. |
| 5 | Give a brief rationale for the methods selected, i.e. for the search strategy, for the method how relevant data was extracted, and for the analysis of the results. |
| 6 | Give a brief description of the limitations of the review, if applicable accompanied by an explanation why the review is considered as valid despite the respective limitation. For instance, if just three articles were summarized, this should be emphasized here and it could be explained (i) that there are no other high-quality data available and (ii) how this review still provides additional value. |
| 7 | ☐ Tick if the quality of evidence for major results of the analysis has been summarized in the manuscript using the GRADE approach (according to Box 2, Guyatt GH et al. GRADE: an emerging consensus on rating quality of evidence and strength of recommendations. BMJ. 2008 Apr 26;336(7650):924-6. ). Otherwise, please provide a brief explanation of the quality of evidence. |

**Reports on Meta-Analyses**

All meta-analyses to be published in the Journal should have clear objectives **related to drug effects** and be based on a thorough, systematic and recent review of the literature. It is recommended to read our 2015 editorial on meta-analyses prior to submission (Fuhr U, Hellmich M. Channeling the flood of meta-analyses. Eur J Clin Pharmacol. 2015 Jun;71(6):645-647). If a review of the literature is part of a review article (and in most cases it is), please also refer to the section on “Review Articles”.

Usually, a meta-analysis is done to determine drug effects on the basis of a number of individual studies providing data on **the same research questions**. Search strategy, selection process and statistical methods used should be described in detail in the manuscript. Beyond the type and design of selected studies, authors should provide details on exposure regarding **the drug(s) examined**, i.e., substance, dose, route of administration, and duration of treatment. Relevant patient characteristics, e.g. demographics, disease state and comedication, should also be described in sufficient detail. To this end, a table describing the characteristics of included studies should be part of the submission. The preferred length of meta-analyses is as described above for original research papers. Extensive tables, e.g. on included (or excluded) studies, should be submitted as Electronic Supplementary Material (ESM) (for detailed information on ESM see also Electronic Supplementary Material in the Instructions for Authors) and will be published online only.

For any report on meta-analyses, the following “Report on meta-analyses submission form” should be filled in. Respective articles not accompanied by this form with entries in all sections will be rejected without further processing.

**Report on Meta-Analyses submission form**

| 1 | ☒ Tick if this is a meta-analysis of clinical trials and a PRISMA flow diagram and checklist has been added to the material submitted (Page et al. The PRISMA 2020 statement: An updated guideline for reporting systematic reviews. PLoS Med. 2021 Mar 29;18(3):e1003583  ☒ Tick if this is a meta-analysis of observational studies and a MOOSE checklist has been added to the material submitted (Stroup et al., JAMA. 2000 Apr 19;283(15):2008-12).  ☐ Tick if none of the two options mentioned above apply and provide an explanation.  Our meta-analysis contains both clinical trials and observational studies. We therefore submit the PRISMA 2020 checklist as requested for reviews involving clinical trials, supplemented by the MOOSE checklist to ensure appropriate reporting of the observational studies. |
| --- | --- |

| 2 | Provide a brief description of the research question and /or the underlying hypothesis addressed by the meta-analysis. If the meta-analysis is part of a data-mining process, please describe and justify.  Argatroban, a direct thrombin inhibitor, disrupts thrombin-induced vasoconstriction, platelet aggregation, and clot formation, thereby enhancing microvascular perfusion. In acute ischemic stroke (AIS) patients with progressive infarction or early neurological deterioration, combining argatroban with antiplatelet therapy may improve neurological outcomes due to its short half-life and favorable safety profile. Although a prior meta-analysis supported its benefit in reducing END and improving functional outcomes, new evidence has since emerged. This updated systematic review and meta-analysis aims to provide the latest synthesis on the efficacy and safety of argatroban plus antiplatelet therapy in AIS. |
| --- | --- |
| 3 | ☒ Tick to confirm that a table describing relevant details of the individual studies included in the meta-analysis is provided. |
| 4 | Explain the main sources of heterogeneity between studies (e.g., different doses, different drugs; different geographic area; different comorbidities) and why the meta-analysis makes sense for the endpoints reported despite this heterogeneity. For instance, if clinical trials with different drugs sharing the same mechanism of action are meta-analyzed regarding specific adverse effects, this could be justified if the adverse effects are related to the mechanism of action as opposed to an off-target effect specific for an individual drug within the class. (NB: this is not the observed heterogeneity in the results of the meta-analysis which is addressed by statistical tests.)  Despite clinical and methodological differences across the included studies, a meta-analysis remains appropriate and informative for synthesizing evidence on the efficacy and safety of argatroban combined with antiplatelet therapy in acute ischemic stroke (AIS). The primary sources of potential heterogeneity include: (1) variations in argatroban dosing regimens and administration protocols; (2) differences in the antiplatelet backbone (single versus dual antiplatelet therapy, or combinations with alteplase in some trials); (3) variations in patient populations, such as stroke severity (mild-to-moderate vs. more severe), stroke subtypes (e.g., large-artery atherosclerosis or branch atheromatous disease), comorbidities, and geographic regions; and (4) differences in outcome definitions and assessment time points (e.g., varying definitions or follow-up durations for early neurological deterioration [END]).  Meta-analysis is still justified for the key endpoints (reduction in END, improvement in functional outcomes, and safety regarding bleeding complications) because all studies evaluated the same core pharmacological intervention, argatroban as a direct thrombin inhibitor. For safety outcomes such as intracranial hemorrhage or major bleeding, the class effect of thrombin inhibition provides a rational basis for pooling, as these risks are mechanistically linked to the anticoagulant action rather than idiosyncratic properties of individual regimens.  A random-effects model was employed to account for expected between-study variability, and subgroup analyses (by intervention and control type) were performed to explore and explain sources of heterogeneity where present. This approach allows for a more precise overall estimate of treatment effect than individual small studies alone, while acknowledging the clinical diversity inherent in real-world AIS management. |
| 5 | List the most recent and/or most similar reports on meta-analyses published previously in the same area.  The most recent report on this topic is: <https://doi.org/10.1007/s12975-025-01357-x>  Our review is an update of the above review. |
| 6 | Explain the additional knowledge generated by the present meta-analysis as compared to the previous reports on meta-analyses mentioned in section 3 and explain why this is important enough to justify publication of another meta-analysis.  Compared with the previous systematic review and meta-analysis, the current study provides several important updates and refinements. First, it incorporates three additional studies, increasing the total from 14 to 17 studies and substantially enlarging the pooled sample size to over 70,000 patients (51,703 in the argatroban group and 18,759 in the control group). This markedly enhances statistical power. Second, while the prior meta-analysis reported a statistically significant improvement in 90-day functional outcomes (mRS 0–2: OR 1.36; mRS 0–1: OR 1.54), the present analysis found no significant difference in mRS 0–2 (RR 1.03, 95% CI 0.97–1.10) and even a slight favor toward the control group for mRS 0–1: RR 1.22. In contrast, it confirms a robust reduction in early neurological deterioration (END) (RR 0.48, 95% CI 0.30–0.75) and a modest but significant improvement in short-term neurological deficit (NIHSS: MD –0.36). Third, safety findings were strengthened by the inclusion of a composite hemorrhage outcome, which showed no significant increase in bleeding risk (RR 1.10, 95% CI 0.98–1.23). Finally, subgroup analyses by specific antiplatelet regimens further clarified that the benefits on END and neurological improvement appear most pronounced when argatroban is combined with dual antiplatelet therapy (DAPT).  The previous meta-analysis may have overestimated long-term functional benefits due to smaller sample size and evolving study quality. By demonstrating consistent reductions in END and short-term neurological improvement without increased hemorrhagic risk, the present meta-analysis provides stronger support for argatroban as a safe adjunctive therapy in selected AIS patients at risk of early deterioration. This refined understanding helps resolve prior uncertainties, informs clinical decision-making in acute settings, and highlights the need for future large-scale RCTs focused on optimal patient selection and dosing. These incremental yet clinically relevant contributions justify the publication of an updated meta-analysis in a rapidly evolving field. |
| 7 | Give a brief rationale for the methods selected, i.e. for the search strategy, for the method how relevant data was extracted, and for the analysis of the results.  The literature search was limited to PubMed and Scopus because these two databases provide the most comprehensive and up-to-date coverage of peer-reviewed clinical studies in neurology and vascular medicine, with substantial overlap in stroke-related publications. This focused approach balances sensitivity and feasibility while minimizing duplication, in accordance with PRISMA 2020 recommendations for systematic reviews.  Statistical analysis employed a random-effects model (DerSimonian–Laird method) to account for anticipated clinical and methodological heterogeneity across studies (e.g., variations in argatroban dosing, antiplatelet backbone, stroke severity, and geographic populations). Risk ratios (RRs) were used for binary outcomes and mean differences (MDs) for continuous outcomes because these effect measures are the most appropriate and interpretable for the respective data types in meta-analyses of both randomized and observational studies. Subgroup analyses were pre-specified to explore potential sources of heterogeneity while maintaining an overall pooled estimate. |
| 8 | Give a brief description of the limitations of the meta-analysis, if applicable accompanied by an explanation why the meta-analysis is considered as valid despite the respective limitation.  This meta-analysis has several limitations. It included both RCTs and observational studies, which introduced heterogeneity and potential confounding. The included studies also varied in ischemic stroke subtypes, definitions and timeframes for early neurological deterioration (END), timing of antiplatelet administration, and most had relatively small sample sizes. These factors limited subgroup analyses by stroke subtype and reduced statistical power for some outcomes.  Despite these limitations, this updated meta-analysis is worth publishing because it provides the largest synthesis to date, incorporating three additional studies and over 70,000 patients. It offers a more nuanced and contemporary picture than the previous meta-analysis by showing consistent reduction in END and improvement in short-term neurological outcomes (NIHSS) with argatroban plus antiplatelet therapy, without an increase in bleeding risk. Importantly, it clarifies that benefits appear most pronounced when argatroban is combined with dual antiplatelet therapy (DAPT). These refined findings help resolve prior uncertainties, provide clinically relevant guidance for acute ischemic stroke management, and highlight specific gaps for future large-scale RCTs. Therefore, this updated evidence synthesis adds meaningful value to the current literature and justifies publication. |

| 9 | ☐ Tick if the quality of evidence for major results of the analysis has been summarized in the manuscript using the GRADE approach (according to Box 2, Guyatt GH et al. GRADE: an emerging consensus on rating quality of evidence and strength of recommendations. BMJ. 2008 Apr 26;336(7650):924-6. ). Otherwise, please provide a brief explanation of the quality of evidence. |
| --- | --- |
