# Supplementary material for: Efficacy and Safety of Argatroban as an Adjunct to Antiplatelet Therapy in Ischemic Stroke: An Updated Systematic Review and Meta-analysis
Source: Eur J Clin Pharmacol. 2026 Oct 22;82(11):275. doi: 10.1007/s00228-026-04203-7 (PMC13690853; doi:10.1007/s00228-026-04203-7)
Supplement: Supplementary file 5 [file 228_2026_4203_MOESM5_ESM.docx]

**Supplementary File**

**Journal: European Journal of Clinical Pharmacology**

**Efficacy and Safety of Argatroban as an Adjunct to Antiplatelet Therapy in Ischemic Stroke: An Updated Systematic Review and Meta-analysis**

Syeda Takreem Fatima ^a^, Ahmed Raza ^a^, Abdullah Javed ^b^, Shabnam Ijaz ^c^, Mishaal Noor ^d^, Syeda Duaa Uzair ^e^, Abdul Raheem Malik ^f^, Rana Ali Mustafa ^a^, Safi Ur Rehman Daim ^g^

^a^ Department of Medicine, Services Institute of Medical Sciences, Lahore, Pakistan.

^b^ Department of Medicine, Allama Iqbal Medical College, Lahore, Pakistan.

^c^ Department of Medicine, Foundation University Medical College, Islamabad, Pakistan.

^d^ Department of Medicine, Rashid Latif Medical College, Lahore, Pakistan.

^e^ Department of Medicine, Dow University of Health Sciences, Karachi, Pakistan.

^f^ Department of Medicine, Continental Medical College, Lahore, Pakistan.

^g^ Carle Foundation Hospital, Illinois, U.S.

**Corresponding author:** Safi Ur Rehman Daim

Carle Foundation Hospital, Illinois, U.S.

[sdaim@illinois.edu](mailto:sdaim@illinois.edu)

**Figure 1: Summary of the risk of bias of RCTs**

**Figure 2: Summary of the risk of bias of NRS**

**Figure 3: Leave-one-out analysis of mRS 0-2**

**Figure 4: Funnel plot showing the publication bias of mRS 0-2**

**Figure 5: Trim and fill analysis for mRS 0-2**

**Figure 6: Leave-one-out analysis of mRS (0-1)**

**Figure 7: Leave-one-out analysis of early neurological deterioration**

**Figure 8: Leave-one-out analysis of change in NIHSS score**

**Figure 9: Forest plot showing the RR of 7-day mortality for argatroban vs control**

**Figure 10: Forest plot showing the subgroup analysis of mRS (0-1) based on the comparison group**

**Figure 11: Forest plot showing the subgroup analysis of mRS (0-2) based on the comparison group**

**Figure 12: Forest plot showing the subgroup analysis of composite haemorrhage based on the comparison group**

**Figure 13: Forest plot showing the subgroup analysis of intracranial haemorrhage based on the comparison group**

**Figure 14: Forest plot showing the subgroup analysis of stroke recurrence based on the comparison group**

**Figure 15: Forest plot showing the subgroup analysis of organ haemorrhage based on the comparison group**

**Figure 16: Forest plot showing the subgroup analysis of change in NIHSS score based on the intervention group**

**Figure 17: Forest plot showing the subgroup analysis of composite haemorrhage based on the intervention group**

**Figure 18:Forest plot showing the subgroup analysis of intracranial haemorrhage based on the intervention group**

**Figure 19: Forest plot showing the subgroup analysis of stroke recurrence based on the intervention group**

**Figure 20: Forest plot showing the subgroup analysis of organ haemorrhage based on the intervention group**

**Supplementary Table 1: Search strings**

| PubMed | ("ischemic stroke"[tiab] OR "cerebral ischemia"[tiab] OR "brain infarction"[tiab] OR stroke[tiab] OR "Ischemic Stroke"[Mesh] OR "Brain Infarction"[Mesh] OR "Stroke"[Mesh])  AND  ("argatroban"[tiab] OR "argatroban hydrate"[tiab] OR "Argatroban"[Mesh])  AND  ("antiplatelet therap*"[tiab] OR "antiplatelet agent*"[tiab] OR "anti-platelet therap*"[tiab] OR "anti-platelet agent*"[tiab] OR "Dual Anti-Platelet Therapy"[Mesh] OR "Platelet Aggregation Inhibitors"[Mesh]) |
| --- | --- |
| Scopus | ALL (("ischemic stroke" OR "cerebral ischemia" OR "brain infarction" OR "Stroke") AND ("argatroban" OR "argatroban hydrate") AND ("antiplatelet therap*" OR "antiplatelet agent*" OR "anti-platelet therap*" OR "anti-platelet agent*")) |
| Google Scholar | ("ischemic stroke" OR "cerebral ischemia" OR “stroke”) AND “argatroban” AND (“antiplatelet” OR "dual antiplatelet" OR “DAPT”) |

**Supplementary Table 2: Timepoints for NIHSS score for included studies**

| **Study ID** | **NIHSS timepoint(s)** | **Change in score** |
| --- | --- | --- |
| Oguro 2018 | Admission; discharge | Admission – Discharge score |
| Wang 2021 | Admission; discharge | Admission – Discharge score |
| Zhou 2020 | Admission; change in NIHSS at day 7 | Change reported (Admission – 7 day score) |
| Li 2022 | Admission; change in NIHSS at day 7 | Change reported (Admission – 7 day score) |
| Sun 2024 | Admission; discharge | Admission – Discharge score |
| Zhang 2024 | Admission; 90 days | Admission – 90 day score |
| Liu 2020 | Admission; change in NIHSS at day 7 | Change reported (Admission – 7 day score) |
| Yan 2025 | Admission; change in NIHSS at day 7 | Change reported (Admission – 7 day score) |
| Chen 2018 | Admission; discharge | Admission – Discharge score |
| Xu 2025 | Admission; discharge | Admission – Discharge score |
| Zhang 2025 | Admission; 7 days | Admission – 7 day score |

**Supplementary Table 3: Results of Subgroup Analysis Based on the Study Design**

| **Outcome** | **Subgroup** | **Studies** | **Pooled estimate (95% CI)** | **P value** | **I^2^ (%)** |
| --- | --- | --- | --- | --- | --- |
| **mRS (0-2)** | RCTs | 5 | RR: 1.08 (1.02 – 1.15) | 0.01 | 17.4 |
|  | Cohort studies | 8 | RR: 1.02 (0.93 – 1.11) | 0.67 | 71.2 |
| **Composite hemorrhage** | RCTs | 2 | RR: 2.17 (1.07 – 4.41) | 0.03 | 0 |
|  | Cohort studies | 7 | RR: 1.08 (0.96 – 1.21) | 0.21 | 0 |
| **mRS (0-1)** | RCTs | 4 | RR: 1.08 (0.86 – 1.35) | 0.49 | 60.6 |
|  | Cohort studies | 3 | RR: 1.51 (1.32 – 1.73) | <0.0001 | 3.7 |
| **END** | RCTs | 2 | RR: 0.43 (0.26 – 0.71) | 0.001 | 0 |
|  | Cohort studies | 5 | RR: 0.47 (0.24– 0.92) | 0.02 | 74.7 |
| **Change**  **In**  **NIHSS score** | RCTs | 4 | MD: 0.40 (-0.72 – 1.51) | 0.48 | 88.8 |
|  | Cohort studies | 7 | MD: 0.64 (-0.07 – 1.36) | 0.07 | 94.2 |
| **Intracranial hemorrhage** | RCTs | 3 | RR: 1.57 (1.06 – 2.35) | 0.02 | 0 |
|  | Cohort studies | 5 | RR: 0.88 (0.50 – 1.54) | 0.64 | 0 |
| **Stroke recurrence** | RCTs | 2 | RR: 0.80 (0.43 – 1.46) | 0.46 | 0 |
|  | Cohort studies | 3 | RR: 0.96 (0.82 – 1.13) | 0.64 | 0 |

RCT: Randomized Controlled Trial; CI: Confidence Interval; RR: Risk Ratio; MD: Mean Difference; mRS: Modified Rankin Scale; END: Early Neurological Deterioration; NIHSS: National Institutes of Health Stroke Scale

**Supplementary Table 4: Results of Subgroup Analysis Based on Risk of Bias Assessment of Studies**

| **Outcome** | **Subgroup** | **Studies** | **Pooled estimate (95% CI)** | **P value** | **I^2^ (%)** |
| --- | --- | --- | --- | --- | --- |
| **mRS (0-2)** | Low risk | 4 | RR: 1.05 (0.96 – 1.16) | 0.28 | 30.4 |
|  | Moderate risk | 4 | RR: 1.06 (0.98 – 1.14) | 0.16 | 65.4 |
|  | High risk | 5 | RR: 0.99 (0.84 – 1.16) | 0.86 | 78 |
| **Composite hemorrhage** | Low risk | 2 | RR: 2.17 (1.07 – 4.41) | 0.03 | 0 |
|  | Moderate risk | 3 | RR: 0.93 (0.70 – 1.25) | 0.64 | 0 |
|  | High risk | 4 | RR: 1.11 (0.97 – 1.26) | 0.12 | 0 |
| **mRS (0-1)** | Low risk | 3 | RR: 1.07 (0.71 – 1.62) | 0.73 | 68.5 |
|  | Moderate risk | 2 | RR: 1.26 (0.85 – 1.89) | 0.25 | 91.5 |
|  | High risk | 2 | RR: 1.28 (1.00 – 1.92) | 0.05 | 45.2 |
| **END** | Low risk | 1 | RR: 0.43 (0.23 – 0.81) | - | - |
|  | Moderate risk | 3 | RR: 0.36 (0.21- 0.60) | 0.0001 | 0 |
|  | High risk | 3 | RR: 0.53 (0.21 – 1.37) | 0.19 | 77.7 |
| **Change**  **in**  **NIHSS score** | Low risk | 3 | MD: 0.30 (-1.30 – 1.90) | 0.71 | 92.1 |
|  | Moderate risk | 3 | MD: 0.46 (0.05 - 0.86) | 0.02 | 53.1 |
|  | High risk | 5 | MD: 0.79 (-0.22 – 1.79) | 0.12 | 95.9 |
| **Intracranial hemorrhage** | Low risk | 3 | RR: 1.57 (1.06 – 2.35) | 0.02 | 0 |
|  | Moderate risk | 1 | RR: 0.88 (0.32 – 2.41) | - | - |
|  | High risk | 4 | RR: 0.88 (0.44 – 1.73) | 0.70 | 0 |
| **Stroke recurrence** | Low risk | 2 | RR: 0.80 (0.43 – 1.46) | 0.46 | 0 |
|  | Moderate risk | 1 | RR: 0.89 (0.61 – 1.30) | - | - |
|  | High risk | 2 | RR: 0.98 (0.83 – 1.16) | 0.81 | 0 |

CI: Confidence Interval; RR: Risk Ratio; MD: Mean Difference; mRS: Modified Rankin Scale; END: Early Neurological Deterioration; NIHSS: National Institutes of Health Stroke Scale

**Supplementary Table 5: Results of Subgroup Analysis Based on Stroke Phenotype**

| **Outcome** | **Subgroup** | **Studies** | **Pooled Estimate (95% CI)** | **P value** | **I^2^ (%)** |
| --- | --- | --- | --- | --- | --- |
| **mRS (0-2)** | Small vessel/ BAD spectrum | 4 | RR: 1.08 (1.00 – 1.17) | 0.051 | 50 |
|  | Large artery atherosclerosis | 3 | RR: 1.07 (0.97 – 1.18) | 0.15 | 84.6 |
|  | Unselected AIS | 5 | RR: 0.96 (0.84 – 1.10) | 0.57 | 61.1 |
|  | Posterior circulation | 1 | RR: 1.04 (0.82 – 1.82) | - | - |
| **Composite**  **hemorrhage** | Small vessel/ BAD spectrum | 3 | RR: 1.93 (0.50 – 7.42) | 0.3 | 0 |
|  | Large artery atherosclerosis | 2 | RR: 1.02 (0.92 – 1.21) | 0.4 | 10.3 |
|  | Unselected AIS | 3 | RR: 1.68 (0.94 – 2.99) | 0.07 | 0 |
|  | Posterior circulation | 1 | RR: 1.10 (0.98 – 1.23) | - | - |
| **mRS (0-1)** | Small vessel/ BAD spectrum | 4 | RR: 1.43 (1.26 – 1.63) | <0.0001 | 19.5 |
|  | Large artery atherosclerosis | 2 | RR: 1.08 (0.90 – 1.29) | 0.42 | 14.5 |
|  | Unselected AIS | 1 | RR: 0.66 (0.40 – 1.07) | - | - |
| **END** | Small vessel/ BAD spectrum | 3 | RR: 0.36 (0.23 – 0.56) | <0.0001 | 0 |
|  | Large artery atherosclerosis | 2 | RR: 0.72 (0.31 – 1.69) | 0.4 | 70.6 |
|  | Unselected AIS | 1 | RR: 0.47 (0.25 – 0.85) | - | - |
|  | Posterior circulation | 1 | RR: 0.22 (0.01 – 3.98) | - | - |
| **Change in**  **NIHSS score** | Small vessel/ BAD spectrum | 3 | MD: 1.06 (0.25 – 1.88) | 0.01 | 58.2 |
|  | Large artery atherosclerosis | 3 | MD: -0.11 (-1.11 – 0.89) | 0.82 | 88 |
|  | Unselected AIS | 4 | MD: 0.91 (-0.21 – 2.02) | 0.11 | 92 |
|  | Posterior circulation | 1 | MD: 0.04 (-0.45 – 0.53) | - | - |
| **Intracranial**  **hemorrhage** | Large artery atherosclerosis | 3 | RR: 0.78 (0.42 – 1.46) | 0.43 | 0 |
|  | Unselected AIS | 5 | RR: 1.57 (1.07 – 2.29) | 0.02 | 0 |

RCT: Randomized Controlled Trial; CI: Confidence Interval; RR: Risk Ratio; MD: Mean Difference; mRS: Modified Rankin Scale; END: Early Neurological Deterioration; NIHSS: National Institutes of Health Stroke Scale

**Supplementary Table 6: Certainty Assessment of RCT-only Subgroup for All Outcomes**

|  | | | | | | | | | | | |
| --- | --- | --- | --- | --- | --- | --- | --- | --- | --- | --- | --- |
| **Certainty assessment** | | | | | | | **Summary of findings** | | | | |
| **Participants (studies) Follow-up** | **Risk of bias** | **Inconsistency** | **Indirectness** | **Imprecision** | **Publication bias** | **Overall certainty of evidence** | **Study event rates (%)** | | **Relative effect (95% CI)** | **Anticipated absolute effects** | |
|  |  |  |  |  |  |  | **With Antiplatelet therapy** | **With Argatroban + Antiplatelet therapy** |  | **Risk with Antiplatelet therapy** | **Risk difference with Argatroban + Antiplatelet therapy** |
| **mRS (0-2) (assessed with: RR)** | | | | | | | | | | | |
| 1730 (5 RCTs) | serious^a^ | not serious | not serious | not serious | none | ⨁⨁⨁◯ Moderate^a^ | 765/1157 (66.1%) | 365/573 (63.7%) | **RR 1.08** (1.02 to 1.15) | 765/1157 (66.1%) | **53 more per 1,000** (from 13 more to 99 more) |
| **Composite hemorrhage (assessed with: RR)** | | | | | | | | | | | |
| 213 (2 RCTs) | not serious | not serious | serious^b^ | serious^c^ | none | ⨁⨁◯◯ Low^b,c^ | 9/105 (8.6%) | 21/108 (19.4%) | **RR 2.17** (1.07 to 4.41) | 9/105 (8.6%) | **100 more per 1,000** (from 6 more to 292 more) |
| **mRS (0-1) (assessed with: RR)** | | | | | | | | | | | |
| 1095 (4 RCTs) | serious^d^ | very serious^e^ | serious^f^ | not serious | none | ⨁◯◯◯ Very low^d,e,f^ | 473/837 (56.5%) | 157/258 (60.9%) | **RR 1.08** (0.86 to 1.35) | 473/837 (56.5%) | **45 more per 1,000** (from 79 fewer to 198 more) |
| **END (assessed with: RR)** | | | | | | | | | | | |
| 749 (2 RCTs) | serious^g^ | not serious | not serious | not serious | none | ⨁⨁⨁◯ Moderate ^g^ | 77/580 (13.3%) | 15/169 (8.9%) | **RR 0.43** (0.26 to 0.71) | 77/580 (13.3%) | **76 fewer per 1,000** (from 98 fewer to 39 fewer) |
| **NIHSS score (assessed with: MD)** | | | | | | | | | | | |
| 1437 (4 RCTs) | serious^h^ | not serious | not serious | serious^i^ | none | ⨁⨁◯◯ Low ^h, i^ | 924 | 513 | 924 | - | MD **0.40 lower** (-0.72 lower to 1.51 higher) |
| **Intracranial hemorrhage (assessed with: RR)** | | | | | | | | | | | |
| 883 (3 RCTs) | not serious | not serious | not serious | not serious | none | ⨁⨁⨁⨁ High | 55/453 (12.1%) | 27/430 (6.3%) | **RR 1.57** (1.06 to 2.35) | 55/453 (12.1%) | **69 more per 1,000** (from 7 more to 164 more) |
| **Stroke recurrence (assessed with: RR)** | | | | | | | | | | | |
| 661 (2 RCTs) | not serious | not serious | not serious | serious^j^ | none | ⨁⨁⨁◯ Moderate^j^ | 22/333 (6.6%) | 17/328 (5.2%) | **RR 0.80** (0.43 to 1.46) | 22/333 (6.6%) | **13 fewer per 1,000** (from 38 fewer to 30 more) |

**CI:** confidence interval; **MD:** mean difference; **RR:** risk ratio

**Explanations**

a. One study (Li 2022) was rated high risk of bias primarily due to concerns in randomization process and outcome measurement; one additional study (LaMonte 2004) had some concerns. So was downgraded one level for serious risk of bias.

b. Included studies differed in comparator (placebo vs DAPT), population, treatment window, and hemorrhage classification systems. These differences introduce meaningful indirectness. Downgraded one level for serious indirectness.

c. The pooled estimate did not reach statistical significance, and the confidence interval includes both no effect and a potentially clinically significant increase in hemorrhagic risk. Downgraded one level for serious imprecision.

d. Li 2022 used a prospective single-arm design with a retrospective historical control without true randomization, constituting high risk of selection bias. Liu 2020 was open-label with a small sample size. Downgraded one level for serious risk of bias.

e. High statistical heterogeneity was detected (I² = 73.9%), with considerable variation in effect estimates across studies. Clinical heterogeneity was also substantial, with studies enrolling different stroke subtypes, different disease severities, and different follow-up structures. Downgraded two levels for very serious inconsistency.

f. Included studies enrolled substantially different stroke populations, and Liu 2020 used argatroban monotherapy rather than argatroban plus DAPT. These differences introduce meaningful indirectness regarding applicability to a general AIS population. Downgraded one level for serious indirectness.

g. Li 2022 used a prospective single-arm design with a retrospective historical control without true randomization, constituting a high risk of selection bias; furthermore, END was defined as ≥1 NIHSS point increase in Li 2022 versus ≥2 points in Xu 2025, introducing measurement inconsistency. Downgraded one level for serious risk of bias.

h. Li 2022 was a prospective, single-arm study where 120 patients receiving the intervention were compared entirely against a historical control cohort collected retrospectively, introducing high risks of selection bias and temporal confounding.

i. While the pooled estimate is tightly bound and has reached strict statistical significance (P = 0.002), the absolute clinical magnitude of the effect is minimal and sits entirely below established clinical relevance thresholds. Downgraded one level for serious clinical imprecision.

j. The pooled estimate demonstrates profound statistical and clinical imprecision. The sample size is heavily underpowered for a rare clinical event like stroke recurrence, and the confidence interval completely fails to exclude either extreme benefit or severe harm. Downgraded one level for serious clinical imprecision.

**Supplementary Table 7: Definitions of END**

| **Sr no.** | **Study ID** | **Definition of END** |
| --- | --- | --- |
| 1 | Nakashima 2025 | Not defined |
| 2 | Xu 2025 | ≥2 points in NIHSS score within 7 days |
| 3 | Zhang 2025 | ≥2 points in NIHSS score within 7 days |
| 4 | Yan 2025 | Not defined |
| 5 | Jin 2024 | Not defined |
| 6 | Adeoye 2024 | (1) An increase of ≥2 points in the total NIHSS score  (2) An increase in the motor score (5a–6b) of ≥1 point  (3) Any new neurological deficit (including deficits that were not measurable by the NIHSS score) |
| 7 | Sun 2024 | Incremental increase in the NIHSS score of: 1- ≥1 point in motor power or 2 - ≥2 points in the total score within 7 days of admission |
| 8 | Zhang 2024 | Not defined |
| 9 | Li 2022 | 2 points or greater increase in NIHSS score at 7 days after  hospitalization |
| 10 | Wang 2021 | ≥1 point increase in NIHSS at 7 days compared with baseline |
| 11 | Xu 2021 | ≥2 point increase in NIHSS within 48 hours after admission |
| 12 | Zhou 2020 | Not defined |
| 13 | Liu 2020 | Not defined |
| 14 | Oguro 2018 | ≥2 point increase in NIHSS within 48 hours from symptom onset |
| 15 | Chen 2018 | Not defined |
| 16 | Wada 2015 | ≥2 point increase in NIHSS within 7 days compared to best post-stroke score |
| 17 | LaMonte 2004 | Not defined |
